# Role of extracellular vesicle-carried ncRNAs in the interactive ‘dialogue’ within the brain and beyond: emerging theranostic epigenetic modifiers in brain-derived nanoplatforms

**DOI:** 10.1186/s40035-025-00502-8

**Published:** 2025-08-05

**Authors:** Nima Sanadgol, Pegah Mousavi, Fatemeh Sadri, Clara Voelz, Miriam Scheld, Roghayeh Khalseh, Javad Amini, Elham Karimi, Amid Rahi, Mohammad-Reza Sepand, Cordian Beyer, Markus Kipp

**Affiliations:** 1 Institute of Neuroanatomy, RWTH University Hospital Aachen, 52074 Aachen, Germany; 2 Endocrinology and Metabolism Research Center, Hormozgan University of Medical Sciences, Bandar Abbas, Iran; 3 Department of Genetics and Molecular Medicine, Faculty of Medicine, Zanjan University of Medical Sciences, Zanjan, Iran; 4 Institute of Functional and Applied Anatomy, Hannover Medical School, Hannover, Germany; 5 Natural Products and Medicinal Plants Research Center, North Khorasan University of Medical Sciences, Bojnurd, Iran; 6 Department of Medical Genetics, Faculty of Medicine, Tehran University of Medical Sciences, Tehran, Iran; 7 Pathology and Stem Cell Research Center, Kerman University of Medical Sciences, Kerman, Iran; 8 Pelotonia Institute for Immuno-Oncology, The Comprehensive Cancer Center-James Cancer Hospital and Solove Research Institute, The Ohio State University, Columbus, USA; 9 Institute of Anatomy, Rostock University Medical Center, Rostock, Germany

**Keywords:** MiRNAs, LncRNA, CirRNA, Exosomes, Epigenetic, Brain

## Abstract

Proper brain function and overall health critically rely on the bidirectional communications among cells in the central nervous system and between the brain and other organs. These interactions are widely acknowledged to be facilitated by various bioactive molecules present in the extracellular space and biological fluids. Extracellular vesicles (EVs) are an important source of the human neurosecretome and have emerged as a novel mechanism for intercellular communication. They act as mediators, transferring active biomolecules between cells. The fine-tuning of intracellular trafficking processes is crucial for generating EVs, which can significantly vary in composition and content, ultimately influencing their fate and function. Increasing interest in the role of EVs in the nervous system homeostasis has spurred greater efforts to gain a deeper understanding of their biology. This review aims to provide a comprehensive comparison of brain-derived small EVs based on their epigenetic cargo, highlighting the importance of EV-encapsulated non-coding RNAs (ncRNAs) in the intercellular communication in the brain. We comprehensively summarize experimentally confirmed ncRNAs within small EVs derived from neurons, astrocytes, microglia, and oligodendrocytes across various neuropathological conditions. Finally, through in-silico analysis, we present potential targets (mRNAs and miRNAs), hub genes, and cellular pathways for these ncRNAs, representing their probable effects after delivery to recipient cells. In summary, we provide a detailed and integrated view of the epigenetic landscape of brain-derived small EVs, emphasizing the importance of ncRNAs in brain intercellular communication and pathology, while also offering prognostic insights for future research directions.

## Introduction

**Fig. 1:**
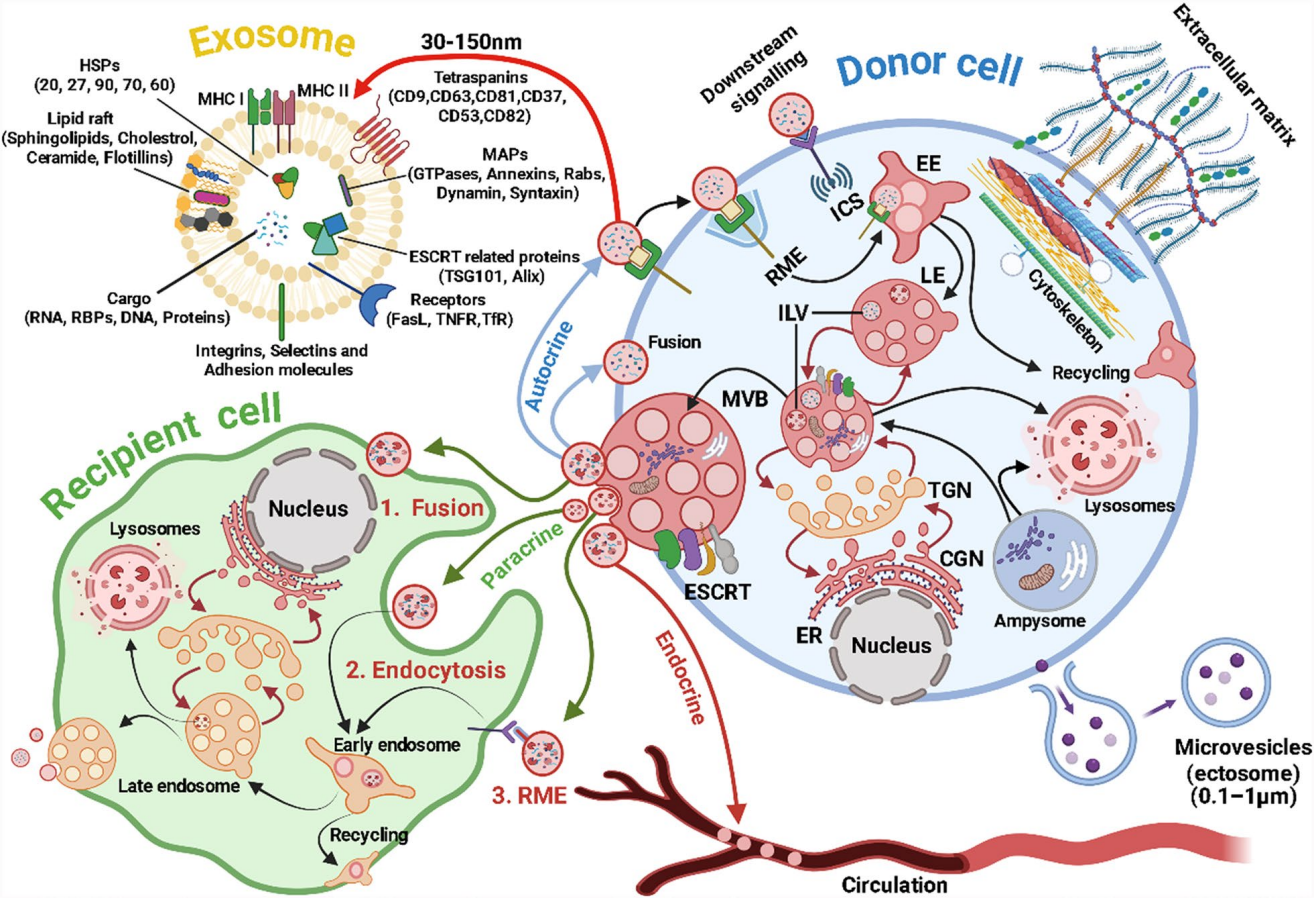
Communications between donor and recipient cells via exosomes. EVs released from donor cells can deliver their contents to recipient cells through three main pathways, including paracrine signaling (EVs are delivered to neighboring cells in the vicinity), endocrine signaling (EVs enter the bloodstream and are transferred throughout the body), and autocrine signaling (EVs are taken up by the same donor cell that released them). In each pathway, EVs can be taken up by recipient cells through fusion (directly merging with the plasma membrane of the recipient cell, and releasing contents into the cell), classic endocytosis (engulfed by the recipient cell through formation of vesicles), or receptor-mediated endocytosis (RME). Exosome content can be highly heterogeneous, including a variety of cargos such as nucleic acids and different kinds of proteins. Figure was created using BioRender (BioRender.com) and is used with permission

Extracellular vesicles (EVs) are tiny spherical structures enclosed by a phospholipid bilayer membrane, which are released by all eukaryotic and prokaryotic cells into the extracellular space. Under physiological conditions, EVs maintain central nervous system (CNS) homeostasis by facilitating communication between CNS cell populations. In response to CNS injury, EVs mediate stress-dependent responses and regulate tissue damage and repair, thereby influencing neurological disease pathogenesis, development, and recovery [1]. Cell-to-cell communication through secreted vesicles, particularly EVs, is a well-documented process in neurobiology. This form of communication facilitates the transfer of molecular signals over short and long distances within the nervous system, like hormones function across various physiological systems. As such, while the fundamental role of secreted vesicles in cell-to-cell communication is well-established in neurobiology, the broader understanding of their functions across different cell types and biological contexts is still evolving. Secreted vesicles, such as exosomes and microvesicles, carry bioactive molecules that influence cellular functions and interactions. Exosomes, which range from 30 to 150 nm in diameter, are formed as intraluminal vesicles (ILVs) through the inward budding of multivesicular bodies (MVBs) within the cell (Fig. 1). These MVBs then fuse with the plasma membrane, releasing exosomes into the extracellular space. Conversely, microvesicles, also known as ectosomes or shedding vesicles, are a larger and very heterogeneous population, varying from 100 to 1000 nm (apart from apoptotic bodies bigger than 1 μm). They are released from the outward budding and fission of the cell's plasma membrane (Fig. 1) [2]. The interaction between the extracellular matrix and the cytoskeleton plays a crucial role in the release and transfer of vesicles into the surrounding tissue (Fig. 1) [3]. The multitude of terms based on function, biogenesis, size, or cell of origin, the diversity of isolation methods and contexts, and the scarcity of validated biomarkers have resulted in numerous misconceptions and sometimes conflicting definitions for EVs in the scientific literature, including terms like supermers, exomers, migrasomes, and oncosomes [4]. In response to this challenge, the International Society for Extracellular Vesicles (ISEV) has called “extracellular vesicle” a generic nomenclature encompassing all kinds of vesicles released by cells. Additionally, the ISEV has published recommendations outlining the minimal requirements for studies focusing on EVs, which are regularly updated [5]. For example, EVs exhibit considerable heterogeneity, especially in cell types where MVBs contain ILVs of diverse sizes and compositions. Consequently, relying solely on features such as size and density as strict criteria for defining EVs is not viable. It is important to be cautious when utilizing isolation procedures and commercially available kits, as well as techniques like electron microscopy, flow cytometry, or nanoparticle tracking, because these methods often struggle to efficiently distinguish between differently sized EVs and membrane-free macromolecular aggregates. Utilizing multiple methods in parallel and employing complementary techniques such as ultracentrifugation, immunoblotting, and mass spectrometry can significantly enhance the quality of a study [6]. On the other hand, inconsistency in the methods used across different studies makes it very difficult to compare their results and decreases the reproducibility of findings.

EVs encompass a wide assortment of molecular cargos comprising proteins, lipids, and various types of nucleic acids, such as DNA, mRNA, and non-coding RNAs (ncRNAs), including microRNAs (miRNAs), transfer RNA, ribosomal RNA, small interference RNAs (siRNAs), circular RNAs (circRNAs) and long ncRNAs (lncRNAs). NcRNAs such as miRNAs, circRNAs, siRNAs, and lncRNAs are essential for regulating gene expression and maintaining cellular function. MiRNAs engage in post-transcriptional regulation by binding to complementary sequences on target mRNAs, leading to their degradation or inhibition of their translation. The siRNAs are vital components of RNA interference, which silence gene expression by degrading mRNA post-transcriptionally. CircRNAs can function as miRNA sponges, modulate transcription, and interact with RNA-binding proteins. LncRNAs participate in various regulatory processes, including chromatin remodeling, transcription, and post-transcriptional modification, thus influencing gene expression at multiple levels. Collectively, these ncRNAs are crucial for genetic regulation and cellular homeostasis [7]. The selective sorting of these cargo molecules into EVs enables them to mirror the physiological condition and source of the originating cell.

Selective ncRNA sorting into EVs is influenced by several mechanisms, including post-transcriptional modifications (e.g., m6A methylation), RNA-binding proteins (RBPs), specific sequence motifs, and cellular stress responses. m6A modifications can enhance ncRNA recognition by RBPs like YTHDF family proteins, facilitating their packaging into EVs. Additionally, tetraspanins, hnRNPs, and AGO2 play crucial roles in sorting miRNAs, while ALIX and endosomal sorting complex required for transport (ESCRT) complexes regulate RNA loading. These mechanisms impact EV functionality, influencing cell-to-cell communication, immune regulation, and disease progression by ensuring the selective transfer of biologically active ncRNAs to recipient cells [8].

Upon release, EVs can traverse body fluids, reaching specific target cells or tissues, where they transfer their cargos, influencing the function of their recipient cells (through either endocrine, paracrine, or autocrine signaling) (Fig. 1). EVs serve a vast range of functions, encompassing intercellular communication, immune modulation, tissue repair, and the transfer of genetic information. Their influence extends to critical processes such as immune responses, cell signaling, angiogenesis, and tissue regeneration [9–11]. EVs in body fluids have gained attention as potential biomarkers for neurological disorders. Compared to existing diagnostic methods such as neuroimaging (MRI, PET scans), cerebrospinal fluid (CSF) analysis, and blood-based biomarkers, EV-based biomarkers offer several advantages, including non-invasive collection, the potential for early detection, and the ability to provide real-time insights into disease progression.

However, challenges remain in standardizing EV isolation and characterization to ensure reproducibility and clinical applicability. While CSF biomarkers (e.g., tau and amyloid-beta [Aβ] in Alzheimer’s disease [AD]) are highly specific, they require invasive lumbar punctures. In contrast, serum or plasma-derived EVs offer a minimally invasive alternative but may suffer from contamination with non-brain-derived EVs. Additionally, neuroimaging techniques provide structural and functional insights but lack molecular specificity, making EV-derived biomarkers a potentially complementary tool rather than a replacement [12]. For example, EV-mediated downstream intercellular signaling has been implicated in amyotrophic lateral sclerosis (ALS), AD, Huntington's disease, Parkinson's disease (PD), and multiple sclerosis (MS) [13–17]. Identifying crucial biomarkers for these conditions is paramount, as it can aid in developing disease-modifying treatments and enhance diagnostic accuracy. Given their diverse components, EVs present promising candidates for biomarker studies related to neuropathological conditions. A thorough analysis of the molecular cargos, particularly ncRNAs, carried by EVs can offer valuable insights into the state of disease responses to drugs and assist in identifying potential biomarkers [18]. Moreover, EVs hold great promise in drug delivery, as they can be engineered to transport specific cargos and precisely target recipient cells [19]. The blood–brain barrier (BBB) acts as a gatekeeper, preventing approximately 98% of therapeutic drugs from entering the CNS. Exosomes can cross the BBB through mechanisms like receptor-mediated transcytosis, adsorptive-mediated transcytosis, and inflammation-driven permeability changes. This ability makes them valuable for intercellular communication, biomarker transport, and potential therapeutic applications in neurological diseases. Their role in drug delivery is being explored, as they can carry molecules across the BBB to target brain disorders. Additionally, brain-derived exosomes may influence immune responses and disease progression by signaling to peripheral cells and transferring ncRNAs [19, 20]. This emerging field has opened up exciting possibilities for advancing diagnostics and therapeutic interventions. Moreover, under pathological conditions, the ncRNA cargos of brain-derived EVs (BDEVs) can profoundly affect neuronal and glial cell function, contributing to disease progression and offering potential therapeutic targets [20].

BDEVs can be categorized into four main groups based on their cellular origin: neuronal-derived extracellular vesicles (NDEVs), astrocyte-derived extracellular vesicles (ADEVs), microglia-derived extracellular vesicles (MDEVs), and oligodendrocyte-derived extracellular vesicles (ODEVs). In this review, we aim to review experimentally validated cell-specific BDEVs and their enriched ncRNAs from various perspectives. During the purification process, larger EVs often remain in the final sample, leading to a mixed population of vesicles, despite a higher concentration of smaller EVs. We carefully use the term “small EVs (sEVs)” instead of “exosomes” in sections of the article where the study's methods cannot precisely identify the enrichment of such sEVs. However, we focus on the ncRNA cargos of sEV-enriched fractions, confirmed with specific protein markers, and collect these data for future evaluations. Subsequently, we use in silico analysis to predict the essential molecular targets associated with these ncRNAs to clarify how they may influence or contribute to pathological conditions.

## Materials and methods

### Search strategy

We employed specific MeSH terms, including “extracellular vesicles” and “exosomes”, “non-coding RNAs”, “nerve cells”, “neurons”, “neuroglia cells”, “astrocytes”, “microglia”, “oligodendrocytes”, and “glioma” as search keywords in PubMed, Scopus, and Web of Science from January 2014 to June 2024. The objective was to acquire relevant articles on nerve/neuroglia cell-derived EVs/exosomes and their ncRNA contents. We merged the titles obtained from the search databases according to the Preferred Reporting Items for Systematic Reviews and Meta-Analyses guidelines. Our review criteria included the following: original articles written in English that focused on EVs/exosomes associated with the CNS and evaluated their ncRNA content. We excluded reviews or other classifications apart from original articles and articles that lacked ncRNA function analysis or did not assess cell-specific EVs or related neuropathologic conditions. After eliminating duplicate articles and thoroughly scanning the remaining ones, we selectively chose articles in which ncRNAs were recognized for their roles in neurological disorders (based on abstract content). Subsequently, we read all full-text manuscripts and excluded those not meeting the inclusion criteria. During the reading process, we extracted the following information from the articles: observed changes in ncRNA expression, relevant disease, type of functional analysis, biological mechanism, source of EVs, and the method used to extract EVs. However, we could not conduct a meta-analysis due to the limited number of studies providing an exact mechanism of action for each ncRNA and the resulting low statistical power (power analysis is conducted using the G*Power 3.1.97 software). For instance, most studies focus on different aspects of ncRNA function, such as their role in gene regulation or involvement in cellular pathways. Variability in experimental methods, such as differences in EV extraction methods, RNA sequencing techniques, or cell models, may hinder comparison between studies.

### In silico analysis

After selecting and compiling experimentally confirmed ncRNAs, we evaluated the functions of human miRNAs, animal miRNAs with 100% sequence similarity to human miRNAs, as well as human lncRNAs and circRNAs with confirmed available sequences, using the following bioinformatic tools. CircAtlas 3.0 (https://ngdc.cncb.ac.cn/circatlas) was used to predict the miRNA targets of circRNAs, utilizing TargetScan, miRanda, and Pita algorithms [21]. LncRNA and miRNA interactions were evaluated using the NPInter v4 database (http://bigdata.ibp.ac.cn/npinter), which compiles ncRNA interactions from CLIP-seq, PARIS, CLASH, ChIRP-seq, and GRID-seq experimentally validated data [22]. The MEINTURNET database (http://userver.bio.uniroma1.it/apps/mienturnet/) was used to predict miRNA gene targets, applying a significance threshold of *P* < 0.05 [23]. To identify cellular pathways influenced by miRNAs, the gene targets of miRNAs were submitted to the Enrichr database (https://maayanlab.cloud/Enrichr/), leveraging real experimental data. Pathways with significant enrichment (*P* < 0.05) were identified and analyzed [24]. Protein–protein interaction (PPI) networks for the gene targets of miRNAs were constructed using the STRING 12 database (https://string-db.org/) based on experimentally validated data. The resulting networks were downloaded and further analyzed with Cytoscape v3.10.1. Our analysis focuses on a curated set of experimentally verified ncRNAs rather than an unbiased, transcriptome-wide dataset. We acknowledge that our findings do not capture the entire range of EV-associated ncRNAs but instead provide insights into those validated in the existing literature. Rather than strictly categorizing ncRNAs as cell-type-specific, we identify them as enriched in certain cell types while acknowledging that they are not exclusively expressed in a single type. Moreover, we cross-referenced our findings with publicly available EV-ncRNA datasets to verify whether the identified miRNAs have been reported across multiple cell types. The EVmiRNA database (https://guolab.wchscu.cn/EVmiRNA/) was used to determine whether BDEV miRNAs overlapped with those reported in other cell-derived EVs. This database presents a detailed compilation of EV-associated miRNAs from various cell types and tissues.

## EV biogenesis and release

Detecting and classifying EVs is challenging. However, EVs can be categorized into distinct subtypes based on their origin and composition. Exosomes and microvesicles (also known as ectosomes or shedding vesicles) are the most extensively studied EV subtypes. Exosomes are persistently released from all cells and display a notable enrichment of protein markers like tetraspanins, heat shock proteins, and ESCRT-related components (Fig. 1). Among them, tetraspanins are crucial in organizing membrane microdomains known as tetraspanin-enriched microdomains by forming clusters and interacting with various transmembrane and cytosolic signaling proteins [25]. The ESCRT is a conserved protein complex present from yeasts to mammals, consisting of approximately 20 proteins grouped into four complexes (ESCRT-0, -I, -II, and -III) along with associated proteins (vacuolar protein sorting 4-A, vesicle trafficking 1, and ALG-2-interacting protein X). While ESCRT-dependent and ESCRT-independent mechanisms play roles in protein trafficking to exosomes and exosome biogenesis, a complete understanding of these processes remains elusive. Specifically, the ESCRT-0 complex recognizes and captures ubiquitinated proteins on the endosomal membrane. ESCRT-I and -II complexes deform the membrane into buds containing the captured cargo, and ESCRT-III is responsible for vesicle scission (Fig. 1). Different members of the ESCRT machinery have been implicated in exosome biogenesis and secretion in various cell types [26]. The tumor suppressor protein p53 and its transcriptional target, tumor suppressor-activated pathway 6, have also been described to regulate exosome secretion, potentially linked to the ESCRT-III component charged multivesicular body protein 1 A [27]. This highlights the potential connections between signaling pathways and exosome biogenesis. Overall, the intricate involvement of the ESCRT machinery and associated proteins in exosome biogenesis underlines the complexity of this process, which still requires further exploration to unravel its precise mechanisms.

## Resource, extraction, and tracking of sEVs

**Fig. 2:**
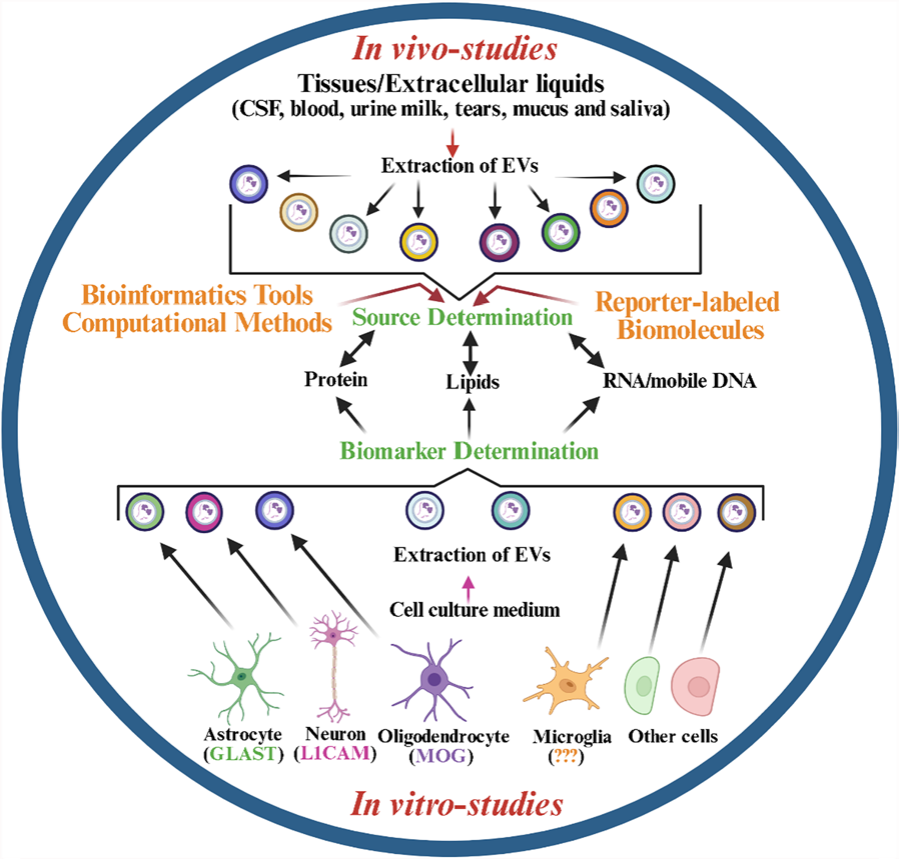
Different methodologies and experiments can be integrated to comprehensively characterize exosomes. The cellular origin of EVs extracted from biological fluids can be determined through bioinformatic and computational analyses, by labeling them in individual cells before release or sorting them according to unique signatures during or after extraction. The signature of cell-specific EVs can be validated in vitro in cultures of primary cells or cell lines. Figure was created using BioRender (BioRender.com) and is used with permission

EVs can be derived from various sources, including tissues, cell culture supernatants, and biofluids like blood, saliva, urine, tears, mucus, CSF, and breast milk [28]. While in vitro studies using tissue and cell supernatant samples are less complicated, biofluids containing a complex mixture of EVs originating from various cell types are more accessible in human studies (Fig. 2). However, working with body fluid samples presents challenges due to the larger starting volumes, leading to dilution issues and low EV yield and purity. This challenge intensifies when isolating cell-type-specific EVs or specific EV subpopulations from biofluids. Therefore, an effective and clinically applicable EV isolation method should address the sensitivity to individual EV subpopulations, purity, throughput, reproducibility, standardization, scalability, and external validity across various clinical settings and samples [29].

There is a wide range of established isolation methods, including ultracentrifugation, density gradient ultracentrifugation, size exclusion chromatography, immunoprecipitation via beads, field-flow fractionation, tangential flow filtration, nanofluidic deterministic lateral displacement, and acoustic trapping technology [30]. Identifying the specific organ source of EVs is complex due to the lack of unique and exclusive markers for each organ. Although no single marker is exclusive to any particular organ-derived EVs (organ-to-organ cross-talk), some markers may still provide clues about the tissue of origin. In the context of brain-derived sEVs, proteins such as growth-associated protein 43 (GAP43), neuroligin 3 (NLGN3), amphiphysin 1, neural cell adhesion molecule, L1 cell adhesion molecule (L1CAM), glutamate aspartate transporter 1 (GLAST-1), and myelin oligodendrocytes glycoprotein (MOG) could be tentatively employed to identify neurons, astrocytes, or oligodendrocytes, respectively [1] (Fig. 2). While these markers are useful for identifying specific cellular origins of sEVs, they may not capture all potential cell types or pathological conditions from which EVs may be derived. Researchers can make educated guesses about the likely source by comparing the protein composition of sEVs to known cell-specific markers [31]. In this regard, markers such as aldehyde dehydrogenase 1 family member L1 (ALDH1L1) and glial fibrillary acidic protein (GFAP) have not yet been evaluated in ADEVs. Moreover, it is recommended that other myelin-related proteins, myelin basic protein (MBP), myelin-associated glycoprotein (MAG), and proteolipid protein (PLP) also be used in parallel with MOG to better characterize ODEVs. Identifying specific markers for MDEVs is indeed challenging, as they share many markers with other myeloid cells, complicating their distinct identification. However, the presence of transmembrane protein 119 (TMEM119) alongside Iba1 (ionized calcium-binding adapter molecule 1) in sEVs offers a promising strategy for considering these vesicles as MDEVs. These two markers are associated with microglial cells, which are key myeloid cells in the brain, suggesting that their co-presence in sEVs could be a valuable indication of microglial origin. While these are key markers for glial cells, they should be used in conjunction with other markers for more precise identification of EV cellular sources in some contexts.

Moreover, sEVs carry various types of RNAs, and some of them may be specific to certain cells. By analyzing the RNA content of sEVs, researchers can also infer their tissue origin or cellular origin [32]. In experimental settings, sEVs can be labeled with isotopes specific to a particular cell or tissue, allowing for tracking the sEVs and identifying their origins (Fig. 2) [33]. Computational methods, such as machine learning algorithms, can be applied to large datasets of sEV profiles to identify patterns associated with specific organs and help predict the most likely tissue source (Fig. 2) [34]. Additionally, utilizing tetraspanin-based pH-sensitive fluorescent reporters for live imaging enables quantification of the fusion rate between MVBs and the plasma membrane at the single-cell level (Fig. 2) [35].

It is important to recognize that identifying the exact cellular source of sEVs in complex biological samples like blood remains challenging. Multiple factors, such as the physiological state and the presence of numerous cell types, can influence the composition of sEVs. In vitro experiments conducted under various culture conditions or with the application of specific cellular stressors significantly influence the composition and release of EVs. This variation complicates the identification of reliable and unique markers for determining their cellular origins, particularly in complex biological samples. For example, changes in the microenvironment, such as nutrient deprivation, hypoxia, or inflammation, can alter the types of EVs released and their molecular cargo. This underscores the importance of selecting specific markers that are stable under the given experimental conditions. In some cases, using specific stressors or manipulating cell cultures to mimic disease conditions can provide more accurate insights into the biological roles of EVs and the cell types they derive from. To further enhance the standardization of research in this field, the EV-TRACK platform has been introduced. This platform aims to facilitate a more systematic reporting of EV biology and methodology, contributing to a clearer and more consistent understanding of this dynamic study area [36]. As research in this field progresses, new techniques and technologies may improve our ability to discern the origin of sEVs more accurately.

## Synaptic vesicles and EVs in neuron communications

**Fig. 3:**
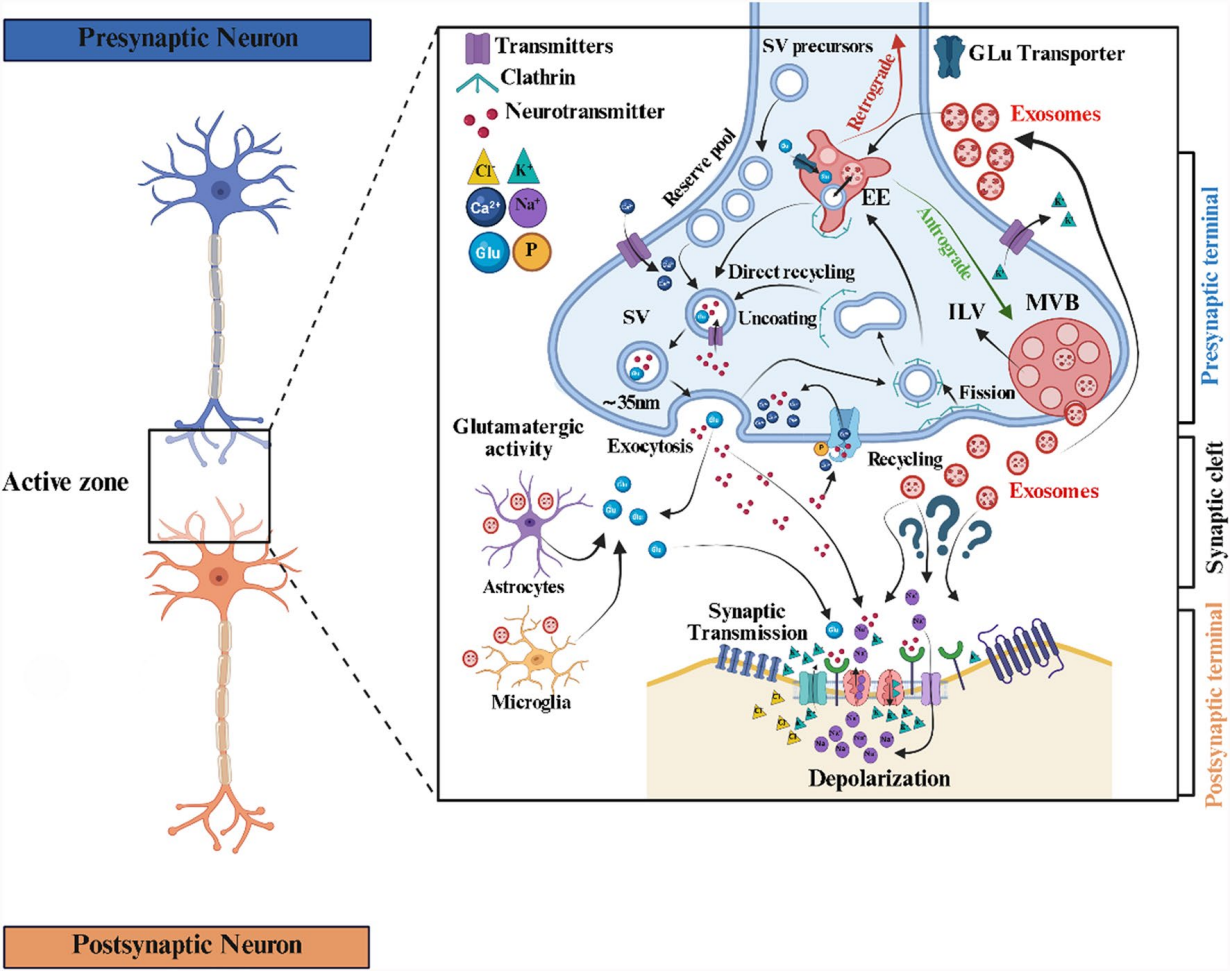
The role of brain-derived exosomes in the synaptic cleft. EVs, such as exosomes, exert various functions within the synaptic cleft, facilitating intercellular communication and potentially impacting synaptic function. They participate in the transmission of signaling molecules, clearance of neurotoxic proteins, modulation of neuroinflammatory responses, and regulation of synaptic development and plasticity. However, their confirmation remains elusive, as depicted by question marks in this illustration. Figure was created using BioRender (BioRender.com) and is used with permission

Both synaptic vesicles (about 40–50 nm) and neuron-derived exosomes (about 30–100 nm) are membrane-bound vesicles falling in the EVs category, involved in cellular communication in the synaptic cleft. Still, they differ in formation, cargo content, and function (Fig. 3). Synaptic vesicles are specific to neurons, formed within the nerve terminals (synaptic boutons), and primarily associated with the release of neurotransmitters into the synaptic cleft during synaptic transmission. The discovery of various RNAs, including ncRNAs, within synaptic vesicles adds another layer of complexity to our understanding of neuronal communication. While synaptic vesicles are traditionally associated with the release of neurotransmitters, the presence of RNA suggests a potential role in the transfer of genetic information between neurons during synaptic transmission [37]. The process of neurotransmission mediated by synaptic vesicles is fundamental for communication between neurons and is essential for various brain functions, including learning, memory, cognition, and behavior. Any disruption in the release, recycling, or reuptake of neurotransmitters via synaptic vesicles can lead to neurological disorders and synaptic dysregulation. NDEVs, on the other hand, originate from the endosomal system of neurons and can influence various cell types within the brain and beyond (Fig. 3) [38]. Calcium influx and the excitability of glutamatergic synapses can influence the release of NDEVs from well-differentiated neurons [39]. Isolating NDEVs through GAP43 (growth-associated protein 43) and NLGN3 immunocapture provides a robust, novel platform for biomarker development in AD. By this method, a study revealed elevated levels of p181-Tau, Aβ42, and neurogranin in NDEVs of AD patients [40]. The interactions of NDEVs with synaptic vesicles remain unknown, but understanding these interactions is crucial for deciphering the brain's complex workings and their impact on overall neurological health.

## EVs in glial cell communications

Glial cells are involved in neuroinflammation and synaptic homeostasis and are essential for maintaining the physiological function of the CNS. Glial cell membranes play a vital role in brain homeostasis by providing ion channels (such as gap junction hemichannels, volume-regulated anion channels, and bestrophin-1), receptors (for neurotransmitters and cytokines), and transporters (like glutamate, glutamate/aspartate, and gamma-aminobutyric acid transporters) [41]. These components are involved in gliotransmission and the regulation of neuronal activity. Numerous studies have indicated that interactions between neurons and glial cells are closely associated with neurological diseases and could potentially be targeted for therapeutic interventions [42]. Therefore, communication between neurons and glial cells is essential for maintaining proper brain function and homeostasis. Recent research has demonstrated that glial cells release EVs carrying various bioactive molecules that travel through the extracellular space and are taken up by neighboring cells, including neurons [43]. The transfer of EVs between neurons and glial cells allows for the exchange of information and signaling molecules**.** These neuron-glia interactions via EVs have been implicated in various physiological processes, including synaptic plasticity, immune responses, and neuroprotection. Dysregulation of these interactions may also contribute to the pathogenesis of neurological disorders and neurodegenerative diseases.

## EV-derived ncRNAs and their epigenetic function

Epigenetic modifications, including DNA methylation, histone modifications, and aberrant expression of ncRNAs, have been validated as active contributors to neurological disorders and promoters of disease progression [44]. EVs, including exosomes or sEVs, contribute significantly to intercellular communication, and their cargos, including ncRNAs, can indeed influence epigenetic regulation in recipient cells [45]. The analysis of exosomal ncRNAs has emerged as an innovative and non-invasive strategy for predicting disease progression [46]. The ncRNAs have been implicated in the development of several neurodegenerative disorders via epigenetic modifications [47]. The ncRNA cargo within circulating sEVs, along with free-circulating ncRNAs, functions as valuable biomarkers for a range of diseases and contributes significantly to critical biological processes, including immune regulation, antigen presentation, and cell-to-cell communication [48]. The miRNA-mediated gene silencing can profoundly affect cellular processes and contribute to epigenetic regulation. The miRNAs encapsulated in sEVs exhibit enhanced stability, allowing them to travel long distances in body fluids without degradation by extracellular nucleases. EVs may also carry proteins or molecules involved in epigenetic modifications. For example, they might transport DNA methyltransferases, histone modifiers, or other regulators of epigenetic processes. Moreover, post-transcriptional modifications may contribute to RNA sorting in EVs [49]. The content of EVs, beyond their quantity, displays distinctions between case and control studies. For example, sequencing of the EV-derived miRNAs from MS patients and healthy controls has revealed diverse signature biomarkers, allowing the differentiation of MS patients and those in different stages of the disease. For instance, nine miRNAs (−15b-5p, −23a-3p, −223-3p, −374a-5p, −30b-5p, −433-3p, −485-3p, −342-3p, −432-5p) have been identified to distinguish relapsing–remitting from progressive conditions [50]. Recently, scientists introduced an updated online database called exoRBase 2.0 (http://www.exoRBase.org), which is a repository of EV-derived ncRNAs such as lncRNAs (*n* = 15,645) and circRNAs (*n* = 79,084) from diverse human body fluids (∼1000 human blood, urine, CSF, and bile samples) analyzed by RNA sequencing (RNA-Seq) method [51]. Unfortunately, this database only provides one brain sample (solid organ), and most data originate from blood samples linked to immune-related disorders. To expand data applicability beyond exoRBase 2.0, we propose integrating multiple databases such as EVAtlas, Vesiclepedia, and miRandola, which include EV-derived ncRNAs from various biofluids. Additionally, incorporating tissue-specific EV datasets (e.g., CSF, urine, tumor-derived EVs) and validating findings using GEO or TCGA transcriptomic data can enhance biological relevance. Experimental approaches like RT-qPCR, RNA-seq, and Nano-flow cytometry can further confirm bioinformatic predictions. Cross-species comparisons with animal models may also help validate evolutionary conservation and translational significance, ensuring a more comprehensive analysis of EV-derived ncRNAs.

## NDEVs

Isolating EVs from the brains of human subjects, especially those with chronic neurological disease, is quite difficult due to the restriction on performing brain biopsies (except brain tumors). Available data are usually derived from postmortem samples (a rather small sample size) in which extensive cell damage has already occurred [52]. Neuroscience research has therefore focused on isolating and evaluating NDEVs, which are easily accessible by plasma or serum sampling. This is performed by first extracting total EVs, usually via differential ultracentrifugation, confirmation through assays including western blotting for transpanins, and then enriching for neuronal-specific markers like L1CAM, tubulin beta 3 class III (TUBB3), synaptosomal-associated protein, and GluR2/3 (glutamate receptor AMPA R2/3), using well-known antibodies [38, 53, 54]. L1CAM (or CD171), a transmembrane cell adhesion molecule involved in neuronal development, is frequently used among the various markers studied. However, its application as a marker for isolating NDEVs from liquid biopsies has been debated, with some evidence questioning its reliability [55]. Attention should also be paid to NDEVs extracted from the brain regions where adult neurogenesis occurs. For example, ongoing neurogenesis (if indeed taking place) and the presence of neuronal stem cells (NSCs) in the sub-ventricular zone (SVZ) of the lateral ventricles and the sub-granular zone of the dentate gyrus in the hippocampus of the adult brain may lead to the extraction of polymorph EVs that can interfere with other EVs [56]. Recent studies focusing on the NSC-derived EVs show that enriched ncRNAs within them contribute to gene regulation in recipient cells, with lower immunogenicity and virtually no possibility of malignant transformation [57]. Therefore, this group of EVs may serve as a safer and more effective therapeutic strategy for neuropathologic conditions. As a new approach, future studies aiming to confirm the neuronal origin of EVs could enrich for at least two ncRNAs, miRNA-9 and miRNA-451a (notable brain-enriched miRNAs), or specific neuronal proteins (TUBB3 and L1CAM) to ensure the accurate selection of NDEVs [58].

### miRNAs in NDEVs

Among all ncRNAs, miRNAs have been more extensively investigated. A recent study has confirmed that miRNA-132-3p and miRNA-212-3p exhibit a significant reduction in L1CAM-captured human plasma EVs derived from AD patients (*n* = **5**), in comparison to high pathological controls (*n* = 5) and cognitively intact, pathology-free controls (*n*** = **5) [58]. In contrast, two other NDEV-associated miRNAs, miR-451a and miR-9-5p, showed no substantial differences in expression levels. Additionally, miRNA-132-3p and miRNA-212-3p are highly enriched in induced pluripotent stem cell-derived human neurons, further supporting their potential role in neuronal function and disease pathology [58]. In AD, interestingly, tau protein has been detected in EVs from conditioned media of human induced pluripotent stem cell-derived neurons, as well as CSF and plasma of patients [59]. Furthermore, NSCs in the SVZ of neonatal CD-1 mice actively generate and release EVs that are highly enriched with specific miRNAs, including members of the miR-9 family, Let-7 family, miR-181 family, miR-26a-2, miR-6236, and miR-5112 [60]. When taken up by microglia, these EVs function as non-canonical microglial morphogens, influencing microglial development, activation, and function. Moreover, these miRNA-enriched EVs have been linked to the regulation of neurodevelopmental processes and are increasingly recognized for their potential contributions to neurodevelopmental disorders [60]. Furthermore, miRNA-124-3p has been identified as a NDEV-associated miRNA that can be transferred to astrocytes, where it plays a crucial role in regulating extracellular glutamate homeostasis. By modulating the expression of glutamate transporter-1 (GLT1), miR-124-3p contributes to synaptic activity and excitatory neurotransmission, ultimately maintaining neuronal function. Importantly, studies have demonstrated a significant reduction in miRNA-124-3p levels in the SOD1^G93A^ mouse model of ALS, suggesting its involvement in disease pathology. The downregulation of miRNA-124-3p in ALS may lead to impairment of GLT1-mediated glutamate clearance, exacerbating excitotoxicity and contributing to neurodegeneration [61].

Moreover, studies utilizing sEVs reporter mice (CD63-GFP^f/f^) have provided valuable insights into the selective enrichment of specific miRNAs in neurons and NDEVs [62]. Several miRNAs, including miR-124-3p, Let-7c-5p, miR-149-3p, and miR-125b-5p, are abundantly expressed in neurons and their secreted EVs, indicating a potential role in intracellular and extracellular regulatory processes. Additionally, certain miRNAs, such as miR-7004-5p, miR-7666-5p, miR-296-5p, and miR-3109-5p, exhibit selective enrichment in NDEVs, suggesting their specific involvement in intercellular communication and signaling pathways unique to the extracellular environment [62]. Additionally, other miRNAs, including miR-669m-5p, members of the miR-466 family, miR-297a-5p, and miR-3082-5p, are found to be significantly enriched within NDEVs, further emphasizing the existence of distinct miRNA signatures associated with neuronal sEV-mediated signaling [62].

Among these miRNAs, miR-124-3p has been shown to modulate astrocytic function upon uptake. Specifically, miR-124-3p enhances the expression of GLT1 by suppressing inhibitory regulatory factors, thereby promoting efficient glutamate clearance from the extracellular space. This mechanism is crucial for maintaining synaptic homeostasis and preventing excitotoxicity, which is implicated in various neurodegenerative disorders [62]. Interestingly, elevated levels of miRNA-21a-5p in NDEVs have been associated with neuroinflammatory responses in the mouse brain, particularly following traumatic brain injury (TBI) [63]. This miRNA mediates neuron-to-microglia communication and contributes to the activation of microglial cells in response to neuronal damage [63]. Mechanistically, miRNA-21a-5p is transferred from injured neurons to microglia via NDEVs. miRNA-21a-5p enhances microglial activation by modulating TLR7 (Toll-like receptor 7) and nuclear factor kappa B (NF-κB) signaling, leading to the release of pro-inflammatory cytokines such as interleukin (IL)-6, tumor necrosis factor α (TNF-α), and IL-1β. This cascade exacerbates neuroinflammation and may contribute to secondary injury processes in TBI [63].

Moreover, whole-transcriptome analysis of serum L1CAM-captured EVs has identified significant alterations in the expression profiles of non-coding RNAs in individuals with autism spectrum disorder (ASD). This study revealed 11 dysregulated miRNAs, with the majority exhibiting downregulation compared to typically developing children of the same age group [64]. The most significantly altered miRNA was PC-5p-139289_26, which was absent in control samples from neurotypical children, indicating a possible ASD-specific regulatory function. Additionally, three other miRNAs (PC-3p-275123_15, PC-3p-38497_124, and PC-5p-149427_24) exhibited increased expression levels in ASD cases. In contrast, seven miRNAs, including hsa-miRNA-193a-5p, showed notable downregulation [64]. Further functional investigations are necessary to determine their specific contributions to ASD, which may ultimately pave the way for novel diagnostic biomarkers and therapeutic targets.

In addition, miR-98 expression in the penumbra region remains elevated on the first day but significantly declines by the third day following ischemic stroke in rats, suggesting its role as an endogenous protective factor post-ischemia [65]. Overexpression of miR-98 inhibits platelet-activating factor receptor-mediated microglial phagocytosis, thereby reducing neuronal death. Additionally, after ischemic stroke, neurons secret EVs carrying miR-98 to microglia, protecting stressed but viable neurons from microglial phagocytosis [65].

The anti-inflammatory effects of hiPSC (human-induced pluripotent stem cell)-derived NSC-derived EVs (hNSC-EVs) on LPS-stimulated microglia are significantly reduced when protein pentraxin 3 (PTX3) or miR-21-5p levels are decreased in the EVs [66]. These findings suggest that hNSC-EVs can effectively modulate proinflammatory human microglia into a non-inflammatory phenotype, highlighting their potential for treating neuroinflammation in neurodegenerative diseases. Moreover, the involvement of PTX3 and miR-21-5p in this process opens new possibilities for enhancing the therapeutic efficacy of hNSC-EVs by overexpressing these factors [66].

Another study revealed that NDEVs facilitate functional recovery in a mouse model of spinal cord injury (SCI) by inhibiting M1 microglia and A1 astrocyte activation. MiRNA profiling identified miR-124-3p as the most abundant miRNA in these sEVs, with MYH9 identified as its downstream target. Furthermore, the PI3K/AKT/NF-κB signaling pathway is involved in the alterations of microglial dynamics caused by sEVs miR-124-3p [67] (Table 1).

**Table 1:** Studies on neuronal-derived extracellular vesicles (NDEVs) and their non-coding RNA cargos in neuropathologic conditions

ncRNA cargo	EV source (ncRNA detection method)	Proposed biological function (selection reason)	Functional evaluation	EV extraction method	Disease relation (~ FC)	References
miR-132-3p	L1CAM-captured human plasma and hiPSCs-derived neurons (microarray with qRT-PCR validation)	Potential diagnostic and theragnostic value (preliminary studies and confirmed group differences)	NC	UCF	AD (9-FC↓)	[58]
miR-212-3p					AD (4-FC↓)	
miR-9-5p					AD (unchanged)	
miR-451a					AD (unchanged)	
miR-181 family (a-1)	Primary CD-1 mice neonatal SVZ neural stem cells (small RNA sequencing)	A non-canonical microglial morphogen (significantly abundant, with confirmed group differences)	Taken up by microglia, in silico, and gain/loss-of-function analysis (for Let-7 family)	DGUC	NDDs (NC↑)	[60]
mir-6236						
mir-5112						
miR-26a-3p						
miR-9 family (−2)						
Let7-family (c-3p)						
miR-124-3p	Primary SOD^193A^ mouse neurons (TaqMan® qRT-PCR)	Regulates extracellular glutamate levels and GLT1 expression (prior literature)	Taken up by astrocytes, gain-of-function, and 3′-UTR luciferase analysis	UCF	ALS (0.6-FC↓)	[61]
miR-7004-5p	Primary CD63-GFP^f/+^ mouse neurons (microarray with TaqMan® qRT-PCR validation)	miR-124-3p up-regulates the GLT1 expression by inhibiting factors (significantly abundant in neurons and confirmed group differences)	Taken up by astrocytes, gain/loss-of-function and 3′-UTR luciferase analysis (for 124-3p)	UCF	NGDs (56-FC↑)	[62]
miR-7666-5p					NGDs (45-FC↑)	
miR-296-5p					NGDs (32-FC↑)	
miR-3109-5p					NGDs (18-FC↑)	
miR-669m-5p					NGDs (2195-FC↑)	
miR-466f					NGDs (1260-FC↑)	
miR-297a-5p					NGDs (1024-FC↑)	
miR-3082-5p					NGDs (512-FC↑)	
Let7c-5p					NGDs (4390-FC↑)	
miR-124-3p					NGDs (4705-FC↑)	
miR-149-3p					NGDs (2896-FC↑)	
miR-125b-5p					NGDs (1176-FC↑)	
miR-21a-5p	Primary mouse neurons (miRNA-seq with qRT-PCR validation)	Neuroinflammatory, activating microglia (significantly abundant, colocalized with MAP2-expressing neurons)	Taken up by microglia	UCF	TBI (2.8-FC↑)	[63]
PC-5p-139289_26	L1CAM-captured human serum (microarray and small RNA-seq with qRT-PCR validation)	A predictive biomarker involved in neuron-mediated glycosylation changes (significantly abundant, with confirmed group differences)	In silico analysis	DGUC	ASD (11-FC↑)	[64]
PC-3p-275123_15					ASD (12-FC↑)	
PC-5p-149427_24					ASD (13-FC↑)	
PC-3p-38497_124					ASD (20-FC↑)	
lncRNA-ENST000532430					ASD (NC↑)	
lncRNA-CAND1.11					ASD (NC↑)	
lncRNA-EPS15L1					ASD (NC↑)	
miR-98	Primary rat neurons (qRT-PCR)	Targets PAFR and prevents neuron phagocytosis (prior literature)	Taken up by microglia, and gain/loss-of-function	UCF	AIS (2-FC↓)	[65]
miR-21a-5p	hiPSCs-derived neurons (qRT-PCR)	Anti-inflammatory effects (prior literature)	Taken up by microglia, and loss-of-function	UCF (Kit)	NGDs (NC↓)	[66]
miR-124-3p	Primary mouse neurons (qRT-PCR)	Promotes functional recovery by targeting MYH9 (significantly abundant, with confirmed group differences)	Taken up by microglia/astrocytes, and gain/loss-of-function and 3′-UTR luciferase analysis	UCF (Kit)	SCI (NC↓)	[67]
lncRNA-POU3F3	L1CAM-captured human plasma (microarray with qRT-PCR validation)	Combined with β-glucocerebrosidase activity (significantly abundant, with confirmed group differences)	NC	Microbeads	PD (2-FC↑)	[68]
CircOGDH	Plasma-derived and primary mouse neurons (RNA-seq with qRT-PCR validation)	A potential therapeutic target for ischemia (significantly abundant, with confirmed group differences)	Loss-of-function, 3′-UTR luciferase, in-silico, and protein interaction analysis	UCF	AIS (54-FC↑)	[69]

UCF, Ultracentrifugation; DGUC, Differential gradient ultracentrifugation; hiPSC, human-induced pluripotent stem cell; NDDs, Neurodevelopmental Diseases; NGDs, Neurodegenerative Diseases; AD, Alzheimer's disease; TBI, Traumatic brain injury; ASD, Autism spectrum disorder; PD, Parkinson's disease; AIS, Acute ischemic stroke; SCI, Spinal cord injury; FC, Fold change compared to control; NC, Not clarified, were included in the bioinformatics analysis as they exhibited 100% similarity with human sequences

### LncRNAs and circRNAs in NDEVs

Analysis of serum L1CAM-captured EVs from ASD patients has identified 1745 differentially expressed lncRNAs compared to age-matched, typically developing children, with most showing downregulation [64]. Remarkably, ENST00000532430, CAND1.11, and EPS15L1 exhibit the most significant expression changes, indicating their potential role in ASD-related molecular mechanisms [64]. A study reported elevated levels of lncRNA-POU3F3 and α-synuclein in L1CAM sEVs, along with reduced glucocerebrosidase (GCase) activity in PD patients compared to controls [68]. These biomarkers showed significant variations based on gender, Hoehn-Yahr (H-Y) stage, and UPDRS-III scores. Importantly, lncRNA-POU3F3 levels in L1CAM sEVs exhibited a strong positive correlation with α-synuclein and a negative correlation with GCase activity in PD patients. Furthermore, L1CAM sEVs, lncRNA-POU3F3 levels, and GCase activity were significantly associated with PD severity, including motor and cognitive impairments. The combined assessment of lncRNA-POU3F3 and α-synuclein levels in L1CAM sEVs, along with GCase activity, effectively distinguished PD patients from healthy controls [68]. Moreover, the neuronal-derived CircOGDH (circular RNA derived from oxoglutarate dehydrogenase) has been identified to be highly expressed in plasma EVs of patients following acute ischemic stroke compared to individuals without cerebrovascular diseases. The interaction between CircOGDH and miRNA-5112 resulted in increased expression of the alpha4 (IV) chain of collagen IV, contributing to neuronal damage. Notably, knockdown of CircOGDH led to a significant improvement in neuronal cell viability under ischemic conditions [69] (Table 1).

## ADEVs

Astrocytes are fundamental to neural function in health and disease. Within neuron-astrocyte networks, their perisynaptic processes act as sensors, responding to neurotransmitters and gliotransmitters through volume transmission. Astrocytes also support neural functions in the CNS and mediate inflammatory responses from microglia. Growing evidence highlights their role in maintaining brain homeostasis in response to pro-inflammatory factors in infectious and neurodegenerative diseases [70]. However, the precise mechanisms of transcellular communication remain unclear. Transcellular communication plays a crucial role in diffuse signaling, where receptor activation is not strictly localized, yet can exert substantial effects on overall brain function. This process modulates neural activity by clearing glutamate and releasing gliotransmitters [70]. Additionally, these processes contribute to regulating the volume of the extracellular space and synaptic coverage [71].

There is a growing understanding of EVs as signal vehicles in the CNS, serving as a mode of non-synaptic communication [72]. ADEVs released into the bloodstream hold the potential as markers for stress-induced diseases and CNS disorders. As noted above, despite their promise as biomarkers, challenges exist in accurately categorizing sEVs and determining their source, which impacts their function [73]. Recent research has identified GFAP-positive EVs from astrocytomas in the blood, which may offer the potential for glioma sub-classification [74]. ADEVs, found in peripheral organs during neuroinflammatory conditions or after brain focal radiation, may serve as biomarkers for various pathological conditions and contribute to brain-to-periphery signaling by targeting peripheral organs [75].

### miRNAs in ADEVs

EVs associated with astrocytes are central to the regulation of neuronal morphology, function, ion homeostasis, and inflammatory response. Dysregulation of ADEVs is implicated in various CNS diseases. A study examining the orbitofrontal cortex of individuals with schizophrenia (SCZ) (*n* = 29), bipolar disorder (BD) (*n* = 26), and unaffected controls (*n* = 25) revealed a significant increase in miR-223 that targets glutamate receptors. Noteworthy, the miR-223 expression was inversely correlated with its target genes, including glutamate ionotropic receptor NMDA-type subunit 2B (GRIN2B) and glutamate ionotropic receptor AMPA-type subunit 2 (GRIA2), suggesting its potential role in inhibiting glutamatergic signaling in psychiatric disorders [76]. Further analyses demonstrated that miR-223 is highly expressed in astrocytes and is actively secreted via EVs, with its expression modulated by antipsychotic drugs [76]. Functional studies revealed that adding miR-223-enriched astrocytic EVs to neuronal cultures led to a significant increase in miR-223 levels, accompanied by a marked reduction in GRIN2B and GRIA2 expression, reinforcing its regulatory role in glutamate receptor signaling. These findings highlight miR-223 as a key astrocytic-derived miRNA that may contribute to synaptic dysfunction in SCZ and BD, particularly in individuals with a history of psychosis [76].

ADEVs play a crucial role in the intercellular transfer of phosphatase and tensin homolog (*PTEN*)-targeting miRNAs and facilitate communication between astrocytes and metastatic tumor cells [77]. Importantly, studies have shown that selectively depleting *PTEN*-targeting miRNAs in astrocytes or inhibiting ADEV secretion restores PTEN expression and effectively suppresses brain metastasis in vivo, highlighting the therapeutic potential of targeting this pathway [77]. Further investigations have identified miR-19a, a microRNA highly enriched in astrocytic EVs, as a key regulator of PTEN expression in brain tumor cells. The uptake of miR-19a-containing astrocytic EVs by metastatic cancer cells leads to PTEN downregulation, thereby enhancing tumor cell survival, proliferation, and colonization within the brain [77]. Studies have demonstrated that the astrocyte expressing the stress-regulated enzyme Aldolase C (Aldo C) releases EVs that are subsequently taken up by hippocampal neurons. Interestingly, the Aldo C-containing EVs exhibit a higher ability to reduce dendritic complexity of developing hippocampal neurons compared to EVs from control astrocytes [78]. Bioinformatics and biochemical analyses further revealed that the elevated Aldo C levels correlate with increased miRNA-26a-5p content in astrocytes and their secreted EVs. Notably, neurons transfected with a miRNA-26a-5p mimic showed reduced expression of proteins involved in neuronal morphogenesis and exhibited morphological changes similar to those induced by Aldo C-containing EVs [78]. These findings highlight that ADEVs loaded with miRNA-26a-5p regulate neuronal morphology and synaptic transmission through specific molecular targets.

Other studies have shown that ADEVs released in response to inflammatory cytokines IL-1β and TNF-α exhibit a significantly altered miRNA profile. Importantly, these ADEVs are enriched with miRNA-125a-5p and miRNA-16-5p, which target neurotrophic receptor tyrosine kinase 3 and its downstream effector B-cell lymphoma 2 (Bcl-2), critical for neurotrophic signaling [79]. The downregulation of these targets in neurons correlates with reduced dendritic growth, decreased dendritic complexity, and diminished neuronal excitability, as evidenced by lower spike rates and burst activity. However, blocking miRNA-125a-5p and miRNA-16-5p prevented these adverse effects, preserving dendritic architecture and neuronal firing patterns [79]. A recent study also indicates that pathogenic ADEVs derived from SOD1^G93A^ astrocytes reduce motor neuron (MN) survival, neurite length, and neurite branching. Among the dysregulated miRNAs in astrocyte EVs, miRNA-155-5p plays a pivotal role in the neurotoxicity, likely through its effects on inflammatory pathways and neuronal survival mechanisms [80]. In another study, ADEVs exhibit significant alterations in their miRNA cargos following astrocyte exposure to brain extracts from TBI mice, with over 20 miRNAs being upregulated. Among them, miRNA-873a-5p has been identified as a key regulator of astrocyte-microglia interactions. Further analysis confirmed that miRNA-873a-5p attenuates microglia-mediated neuroinflammation and improves neurological deficits post-TBI by inhibiting the NF-κB signaling pathway. These findings highlight the therapeutic potential of miRNA-873a-5p in modulating neuroinflammation and promoting recovery in TBI patients [81]. Furthermore, ADEVs enriched with miR-200a-3p prevent cell death in 1-methyl-4-phenylpyridinium (MPP⁺)-treated SH-SY5Y cells and glutamate-treated hippocampal neurons through down-regulation of mitogen-activated protein kinase kinase 4 (MKK4), reinforcing its role in mitigating neurotoxicity in in vitro PD models [82]. miRNA target analysis and reporter assays confirmed that miR-200a-3p directly targets MKK4 by binding to two distinct sites on the 3′-UTR of Map2k4/MKK4 mRNA. Treatment with a miR-200a-3p mimic effectively suppressed MKK4 mRNA and protein expression, leading to reduced cell death in MPP-treated SH-SY5Y cells and glutamate-treated hippocampal neuron cultures [82].

The regulatory role of ADEVs in modulating neuronal autophagy has been extensively investigated. To model ischemic injury, the mouse hippocampal neuronal cell line (HT-22) was cultured under oxygen and glucose deprivation (OGD) conditions. Remarkably, ADEVs facilitate the transfer of miRNA-190b, which plays a neuroprotective role by inhibiting OGD-induced apoptosis and autophagy in neuronal cells [83]. Mechanistically, miRNA-190b exerts its protective effects by targeting autophagy-related 7 (ATG7), a key regulator of the autophagic process. By suppressing ATG7, ADEV-loaded miR-190b effectively reduces autophagy activation in response to ischemic stress, thereby preventing excessive neuronal cell death. These findings highlight the potential therapeutic significance of ADEVs in modulating neuronal survival pathways and suggest that miRNA-190b could serve as a promising target for neuroprotection in ischemic brain injury (IBI) [83]. Moreover, ADEVs play a neuroprotective role by transporting miRNA-17-5p, which has been shown to mitigate hypoxic-ischemic brain injury (HIBI) in neonatal rats. In in vivo and in vitro models, ADEVs enriched with miR-17-5p improved neurobehavioral performance and decreased cerebral infarct size, neuronal apoptosis, oxidative stress, and inflammation. Mechanistically, miR-17-5p binds to BCL2-interacting protein 2 (BNIP2) mRNA, leading to its downregulation in OGD cells, thereby enhancing neuronal survival. Furthermore, overexpression of miR-17-5p in H19-7 hippocampal neurons significantly increased cell viability and reduced OGD-induced apoptosis, oxidative stress, and inflammation. Conversely, BNIP2 overexpression reversed these protective effects. These findings highlight the miR-17-5p/BNIP2 axis as a potential therapeutic target for mitigating HIBI [84].

### LncRNAs in ADEVs and glioma-derived extracellular vesicles (GDEVs)

ADEVs enriched with NF-κB interacting long non-coding RNA (NKILA) exert a neuroprotective effect. This protection is facilitated by the interaction between the lncRNA NKILA and miRNA-195, which typically suppresses autophagy by targeting the nod-like receptor X1 (NLRX1). The regulatory crosstalk within the NKILA/miRNA-195/NLRX1 axis significantly influences cellular response mechanisms, thereby contributing to neuronal protection following TBI [85]. In the glioma microenvironment, considerable evidence shows active communication between tumor cells and their surroundings via sEVs, influencing key glioma characteristics [86]. For example, Bian and colleagues uncovered a key mechanism by which GDEVs drive astrocyte activation. They found that GDEVs transport lncRNA activated by transforming growth factor beta (lnc-ATB) to astrocytes, facilitating malignant cell invasion and migration. Importantly, lnc-ATB downregulates miR-204-3p in an Argonaute 2-dependent manner. This suppression leads to astrocyte activation, which, in turn, enhances the migratory and invasive potential of glioma cells. These findings highlight the crucial role of lncRNA-ATB in shaping the glioma microenvironment through sEV-mediated intercellular communication, providing new insights into glioma progression and potential therapeutic targets [87].

Furthermore, Dai et al. discovered that GDEVs overexpressing lncRNA AHIF, which is the natural antisense transcript of hypoxia-inducible factor-1α (HIF-1α), contribute to glioblastoma progression and resistance to radiation therapy. Further biochemical analysis revealed that the lncRNA AHIF regulates factors linked to migration and angiogenesis in sEV, highlighting its involvement in tumor development and adaptation to hypoxic conditions [88]. Similarly, Qiu et al. reported that sEVs enriched with lncRNA-AHIF are significantly upregulated in the serum of patients with endometriosis. Their findings suggest that this lncRNA plays a crucial role in promoting angiogenesis, a key process associated with the progression of endometriosis [89].

Other studies have highlighted the crucial roles of lncRNA colon cancer-associated transcript 2 (lncRNA-CCAT2) and lncRNA POU Class 3 Homeobox 3 (lncRNA-POU3F3) in glioma-associated angiogenesis. GDEVs can transfer lncRNA-CCAT2 to endothelial cells, where it promotes angiogenesis by activating vascular endothelial growth factor A (VEGFA) and transforming growth factor beta (TGFβ). Functional studies further demonstrated that lncRNA-CCAT2 overexpression in human umbilical vein endothelial cells (HUVECs) upregulates VEGFA and TGFβ, increases Bcl-2 expression, and inhibits Bax and caspase-3, thereby reducing apoptosis. Conversely, downregulation of lncRNA-CCAT2 has the opposite effect [90]. Similarly, GDEVs facilitate angiogenesis by transferring lncRNA-POU3F3 to endothelial cells. Human brain microvascular endothelial cells (HBMECs) can efficiently internalize exosomes derived from A172 cells (A172-Exo), resulting in a significantly higher level of lncRNA-POU3F3 compared to cells treated with shA172-Exo. Functionally, A172-Exo exhibited better activity in enhancing HBMEC migration, proliferation, and tubular-like structure formation in vitro, as well as arteriole formation in vivo [91]. These findings underscore the critical role of GDEV lncRNAs in modulating the tumor microenvironment and promoting angiogenesis.

Moreover, lncRNA sequencing in murine astrocytes identified sEV lncRNA 4933431K23Rik (lncRNA 49Rik) as a key regulator in mitigating TBI-induced microglial activation both in vitro and in vivo, ultimately improving cognitive function [92]. Integrated miRNA and mRNA sequencing, along with binding prediction analysis, demonstrated that the sEV lncRNA 49Rik upregulates E2F7 and TFAP2C expression by sponging miR-10a-5p. As transcription factors, E2F7 and TFAP2C further modulate Smad7 expression in microglia. Adeno-associated virus-mediated overexpression of Cx3cr1-Smad7 in microglia effectively suppressed neuroinflammation and alleviated cognitive impairment following TBI [92]. Mechanistically, overexpressed Smad7 directly binds with IκBα, inhibiting its ubiquitination and preventing NF-κB signaling activation [92]. These findings highlight the therapeutic potential of lncRNA 49Rik in sEVs in modulating neuroinflammatory responses and mitigating cognitive impairment following TBI (Table 2).

**Table 2:** Studies that specifically evaluated ADEVs and GDEVs as well as their non-coding RNA cargos in neuropathologic conditions

ncRNA cargo	EV source (ncRNA detection)	Proposed biological function (selection reason)	Functional evaluation	EV extraction method	Disease relation (~ FC)	References
miR-223-3p	Primary mouse cortical astrocytes (NanoString with TaqMan® qRT-PCR validation)	Targets glutamate receptors (significantly abundant, confirmed group differences)	Taken up by neurons, gain-of-function analysis	UCF	SCZ and BD (8-FC↑)	[76]
miR-19a	Primary mouse astrocytes (TaqMan® qRT-PCR)	Decreases PTEN expression (prior literature, significantly abundant, confirmed group differences)	Taken up by tumor cells, in silico, and gain-of-function analysis	UCF	Brain cancer (3.5-FC↑)	[77]
miR-26a-5p	Primary rat astrocytes (TaqMan® qRT-PCR)	Regulates astrocytic enzyme Aldolase C (prior literature, significantly abundant, confirmed group differences)	Taken up by neurons, in silico, and gain/loss-of-function analysis	UCF	NGDs (50-FC↑)	[78]
miR-23a	Primary rat astrocytes (microarray with TaqMan® qRT-PCR validation)	Increased by IL-1β; 125a-5p and 16-5p target NTKR3 and its downstream effector Bcl2 (significantly abundant, with confirmed group differences)	Taken up by neurons, in silico, gain/loss-of-function, and 3′-UTR luciferase analysis (for 125a-5p and 16-5p)	UCF	NID (unchanged)	[79]
miR-16-5p					NID (2-FC↑)	
Let7f-3/5p					NID (3-FC↑)	
miR-100					NID (2.5-FC↑)	
miR-125a-5p					NID (3.5-FC↑)	
miR-125b-5p					NID (2-FC↑)	
miR-23a		Increased by TNFα; 125a-5p and 16-5p targeted NTKR3 and Bcl2 (significantly abundant, with confirmed group differences)			NID (unchanged)	
miR-16-5p					NID (2-FC↑)	
miR-145					NID (3.5-FC↑)	
miR-107-3/5p					NID (2.5-FC↑)	
miR-23a		Increased by ATP (significantly abundant, with confirmed group differences)			NID (unchanged)	
miR-532-5p					NID (2.5-FC↑)	
miR-544-3p					NID (4.5-FC↑)	
miR-21a-5p					NID (2.5-FC↑)	
miR-598-5p					NID (3.5-FC↑)	
miR-501-5p					NID (2.5-FC↑)	
miR-155-5p	Primary SOD1^G93A^ rat astrocytes (qRT-PCR)	Contributes to motor neuron death (prior literature, significantly abundant, confirmed group differences)	Taken up by motor neurons, in silico, and loss-of-function analysis	UCF	ALS (3.5-FC↑)	[80]
miR-582-3p					ALS (2-FC↑)	
miR-873a-5p	Primary mouse astrocytes (microarray)	Inhibits NF-κB pathway (preliminary studies and confirmed group differences)	Taken up by microglia, in silico, and gain-of-function analysis	UCF	TBI (0.5–1.5-FC↑)	[81]
miR-1224-5p		NC	NC			
miR-708-5p						
miR-383-5p						
miR-218-2-3p						
miR-551b-3p						
miR-873a-3p						
miR-219a-2-3p						
miR-128-1-5p						
miR-128-3p						
miR-124-3/5p						
miR-544-5p						
miR-7240-5p						
miR-137-3/5p						
miR-138-5p						
miR-7055-3p						
miR-382-3p						
miR-3099-5p						
miR-200a-3p	Primary mouse astrocytes (small RNA-seq with qRT-PCR validation)	Decreased by MPP, prevents apoptosis via down-regulation of MKK4 (significantly abundant, confirmed group differences)	Taken up by SH-SY5Y cells and neurons*, *in silico, and gain/loss-of-function and 3′-UTR luciferase analysis	UCF	PD (1.79-FC↓)	[82]
miR-150-5p		Expression affected by MPP	NC		PD (1-FC↓)	
miR-138-5p					PD (1-FC↓)	
miR-222-3p					PD (2.27-FC↑)	
miR-423-3p					PD (2.17-FC↑)	
miR-182-5p					PD (2.07-FC↑)	
miR-190b-5p		Inhibits oxygen and glucose deprivation-induced autophagy and neuronal apoptosis by targeting Atg7 (prior literature)	Taken up by HT-22 cells and hippocampal neurons, gain/loss-of-function and 3′-UTR luciferase analysis	UCF	IBI (3-FC↑)	[83]
miR-17-5p	Primary rat cortical astrocytes (qRT-PCR)	Reduces neuronal oxidative stress by suppressing BNIP-2 expression (prior literature)	Taken up by H19-7 cells, injected into the HIBD rat model, gain/loss-of-function, 3′-UTR luciferase, and RNase treatment analysis	UCF	HIBD (2-FC↑)	[84]
lncRNA-NKILA	Human brain astrocytes (qRT-PCR)	Alleviates injury by binding to miR-195 and upregulating NLRX1 (prior literature)	Taken up by neurons, gain/loss-of-function, 3′-UTR luciferase, and RIP analysis	UCF	TBI (NC↓)	[85]
lncRNA-ATB	HGCL (A172 and U251) (qRT-PCR)	Suppresses miR-204-3p in an Ago2-dependent manner (prior literature)	Taken up by astrocytes, gain-of-function, 3′-UTR luciferase, and RIP analysis	UCF (Kit)	Glioma (2.5/4.5-FC↑)	[87]
lncRNA-aHIF	HGCL (U87-MG, U251-MG, A172 and T98G) (qRT-PCR)	Promotes radioresistance by targeting HIF-1α (prior literature)	Gain/loss-of-function analysis	UCF (Kit)	Glioma (NC↑)	[88]
lncRNA-CCAT2	HGCL(U87-MG, U251-MG, A172 and T98G) (qRT-PCR)	Promotes angiogenesis via activation of VEGFA and TGFβ (prior literature)	Taken up by HUVECs, gain/loss-of-function analysis	UCF	Glioma (9-FC↑)	[90]
lncRNA-POU3F3	HGCL(U87-MG, U251-MG, A172 and T98G) (qRT-PCR)	Regulates glioma angiogenesis (prior literature)	Taken up by HBMECs, gain/loss-of-function analysis	UCF	Glioma (6.5-FC↑)	[91]
lncRNA-49Rik	Primary mouse astrocytes (RNA-seq with qRT-PCR validation)	Up-regulates E2F7 and TFAP2C expression by sponging miR-10a-5p (significantly abundant, with confirmed group differences)	Taken up by microglia, gain/loss-of-function, and 3′-UTR luciferase	UCF (Kit)	TBI (5-FC↑)	[92]
circSHOC2	Primary mouse astrocytes (qRT-PCR)	Suppresses apoptosis by acting on the miR-7670-3p/SIRT1 axis (prior literature)	Taken up by neurons, gain/loss-of-function, and 3′-UTR luciferase, and RIP analysis	UCF	IBI (2.5-FC↑)	[93]
circRNA-ATP8B4	U-251MG cells (qRT-PCR)	Promotes radioresistance by sponging miR-766 (prior literature)	In silico analysis	UCF	Glioma (2.5-FC↑)	[94]
circ_0012381	HGCL(U87-MG, U251-MG) (RNA-seq with qRT-PCR validation)	Induces M2 polarization by sponging miR-340-5p to increase ARG1 expression (significantly abundant, confirmed group differences)	Taken up by microglia, gain/loss-of-function, in silico*,* and 3′-UTR luciferase analysis	UCF (Kit)	Glioma (2.5-FC↑)	[95]

UCF, Ultracentrifugation; DGUC, Differential gradient ultracentrifugation; NGDs, Neurodegenerative Diseases; PD, Parkinson's disease; TBI, Traumatic brain injury; ALS, Amyotrophic lateral sclerosis; HGCL, Human glioma cell lines; SCZ, Schizophrenia; BD, Bipolar disorder; NID, Neuroinflammatory diseases; IBI, Ischemic brain injury; ~ FC, approximately Fold change expression compared to control; HIBD, Hypoxic-Ischemic Brain Damage; NC, Not clarified, The highlighted wells were included in the bioinformatics analysis as they exhibited 100% similarity with human sequences

### circRNAs in ADEVs

A recent study reported that circSHOC2, a circRNA derived from ischemic-preconditioned ADEVs, acts as a neuroprotective agent in ischemic stroke by inhibiting neuronal autophagy [93]. SHOC2 protein plays a key role in modulating the ERK1/2 (extracellular signal-regulated kinase) pathway by forming a holophosphatase complex that activates RAF (rapidly accelerated fibrosarcoma) protein. In an in vitro oxygen–glucose deprivation model and an in vivo mouse middle cerebral artery occlusion model, delivery of circSHOC2-enriched EVs improved cellular viability, reduced infarct volume, and alleviated neurobehavioral deficits. Further analysis revealed that the sEV circSHOC2 could be transferred to neurons, inhibiting apoptosis and promoting autophagy by sponging miRNA-7670-3p, increasing sirtuin 1 (SIRT1) levels. Notably, these protective effects were specific to ischemic conditions rather than normal physiological states [93]. In a separate study, another circRNA, circRNA-ATPase phospholipid transporting 8 B4 (circRNA-ATP8B4), was found in radioresistant GDEVs and contributed to glioma radioresistance by acting as a competitive endogenous RNA for miRNA-766. The circRNA-ATP8B4 is transferred from radioresistant EVs to normal glioma U251 cells, where it functions as a miR-766 sponge, ultimately promoting cell survival under radiation exposure [94]. These findings highlight the significant role of sEV circRNAs in neuroprotection and glioma adaptation under pathological stress conditions.

Moreover, radiation-exposed GDEVs significantly promote M2 microglia polarization, which, in turn, enhances the proliferation of irradiated glioblastoma cells [95]. The circ_0012381 is significantly upregulated in irradiated glioblastoma cells and transferred to microglia via sEVs. Within microglia, circ_0012381 facilitates M2 polarization by sponging miR-340-5p, leading to increased ARG1 expression. These M2-polarized microglia exhibit reduced phagocytic activity and support glioblastoma cell growth through the CCL2/CCR2 signaling axis. Importantly, compared to radiotherapy alone, combined inhibition of sEV release significantly suppressed glioblastoma growth in a zebrafish model [95] (Table 2).

## MDEVs and their ncRNA content

Microglia, immune cells in the CNS, play pivotal roles in the onset and progression of neuropathologic conditions. Microglia manifest two primary polarized forms: pro-inflammatory M1 and anti-inflammatory M2. The M1 microglia express, among other inflammatory mediators, IL-6, IL-1, and TNF-α, while the M2 phenotype microglia release IL-4, IL-10, and TGFβ. Recent studies suggest that the activation state of microglia shapes the composition of MDEVs, regulating neuronal function, activating other cells, and controlling cell differentiation [96, 97]. In neuropathologic conditions, microglia, with a dual protective-detrimental role, utilize EVs to modulate various brain cells, including resident, infiltrating, and tumor cells, through long-range diffusion in the CNS. MDEVs carry proteins typically found in late endosomes, strongly suggesting that they originate from sEVs. Furthermore, MDEVs display major histocompatibility complex class II molecules, transpanins, Flotillin 1, and monocyte/macrophage marker CD14, which is often used for characterization [98].

### miRNAs in MDEVs

A recent study demonstrated significant alterations of miRNA-124-3p levels in MDEVs at different stages (acute, sub-acute, and chronic) following repetitive mild TBI [99]. In a mouse model of TBI, intravenously injected microglial sEVs were selectively taken up by neurons in the injured brain. Notably, miR-124-3p was successfully transferred from these sEVs into hippocampal neurons, where it alleviated neurodegeneration through modulation of the Rela/Apolipoprotein E (ApoE) signaling pathway. Additionally, in vitro studies further confirmed the protective effects of microglial EVs enriched with miRNA-124-3p. Neurons subjected to repetitive scratch injuries exhibited reduced neurodegeneration when treated with these sEVs. Mechanistically, miRNA-124-3p targets RelA, a transcription factor known to inhibit ApoE. Since ApoE plays a crucial role in promoting the breakdown of amyloid-beta (Aβ), its upregulation following miR-124-3p intervention leads to reduced Aβ accumulation and associated neurotoxicity [99].

Moreover, increased levels of miRNA-155 were detected in MDEVs following heat stroke, suggesting a critical role of miRNA-155 in mediating neuronal response to thermal stress. Upon transfer into neurons, these miRNA-155-enriched EVs induce neuronal autophagy by directly targeting Ras homolog enriched in the brain (Rheb), a key activator of the mammalian target of rapamycin complex 1 (mTORC1) signaling pathway [100]. By suppressing Rheb expression, miRNA-155 effectively inhibits the mTORC1 activity, which is a central regulator of cell growth, survival, and autophagy. This disruption of mTORC1 signaling results in excessive autophagy activation, leading to neuronal stress and potential neurodegeneration [100].

Recent transcriptional analysis of MDEVs derived from LPS-activated microglia identified a significant upregulation of miRNA-615-5p. miRNA-615-5p specifically binds to the 3′UTR of myelin regulatory factor (MYRF), a crucial transcription factor responsible for regulating myelination in oligodendrocyte lineage cells [101]. Mechanistically, EVs released from activated microglia facilitate the transfer of miRNA-615-5p into oligodendrocyte precursor cells (OPCs). After internalization, miRNA-615-5p directly targets MYRF, resulting in inhibition of OPC differentiation and maturation [101].

Research has identified that ethanol exposure triggers neuroimmune pathology by promoting the release of let-7b/high-mobility group box 1 (HMGB1) complexes within MDEVs [102]. These EVs facilitate the transfer of let-7b/HMGB1 to neighboring neurons, contributing to hippocampal neurodegeneration. This highlights a potential link between chronic alcohol consumption and neuroinflammation, which may be critically involved in the progression of alcohol-related brain damage and the pathology of alcoholism [102].

Inflammatory microglia release EVs enriched with miR-146a-5p, which regulates key synaptic proteins [103]. This microglia-enriched miRNA, absent in hippocampal neurons, suppresses presynaptic synaptotagmin-1 (Syt1) and postsynaptic neuroligin-1 (Nlg1) in neurons, impairing synaptic stability. Blocking EV–neuron interaction or using EVs depleted of active miR-146a-5p prevents this effect, highlighting the role of miR-146a-5p in neuroinflammation and synaptic dysfunction [103]. Moreover, miR-146a-5p transferred via MDEVs reduces the frequency and amplitude of excitatory postsynaptic currents while decreasing dendritic spine density [104]. Additionally, overexpression of miR-146a-5p in the hippocampal dentate gyrus (DG) suppresses neurogenesis and reduces excitatory neuron activity by directly targeting Krüppel-like factor 4 (KLF4). Conversely, downregulation of miR-146a-5p restores adult neurogenesis in the DG and alleviates depression-like behaviors in rats. Interestingly, circRNA ANKS1B functions as a miRNA sponge, sequestering miR-146a-5p and modulating KLF4 expression, thereby regulating neurogenesis and depression-like behaviors [104].

MDEVs also transmit signals to cancer cells, altering glioma metabolism by reducing lactate, nitric oxide, and glutamate (Glu) release [105]. Additionally, MDEVs influence Glu homeostasis by upregulating the expression of Glu transporter Glt-1 in astrocytes. These effects are primarily driven by miR-124 contained within MDEVs. In vivo studies demonstrate that MDEVs significantly reduce tumor mass and prolong survival in glioma-bearing mice, with miR-124 playing a crucial role in these therapeutic effects [105].

Moreover, miR-124-3p in MDEVs promotes anti-inflammatory M2 polarization of microglia, suppresses neuronal inflammation, and enhances neurite outgrowth after injury [106], by inhibiting the mTOR signaling pathway through direct targeting of PDE4B. In a repetitive traumatic brain injury (rTBI) model, miR-124-3p improved neurological outcomes, reduced neuroinflammation, and decreased neurodegenerative markers (Aβ-peptide and p-Tau), highlighting its potential as a therapeutic target for brain injury recovery [106]. sEVs derived from M2-polarized microglia (BV2-Exo) are taken up by neurons and provide neuroprotection by reducing neuronal apoptosis following ischemic injury, both in vitro and in vivo [107]. In ischemic mice, BV2-Exo administration decreased infarct volume and improved behavioral outcomes. Importantly, miRNA-137 was enriched in BV2-Exo and contributed to its neuroprotective effects, with Notch1 as a direct target of miRNA-137 [107]. Moreover, microRNA-151-3p is highly enriched in MDEVs and mediates their neuroprotective effects in SCI repair. Luciferase activity assays identified P53 as a direct target of miR-151-3p, suggesting the involvement of the p53/p21/CDK1 signaling cascade in the regulation of neuronal survival and axonal regrowth by MDEV miR-151-3p [108]. Furthermore, M2-EVs reduce brain atrophy, enhance functional recovery, and promote oligodendrogenesis and white matter repair in vivo [109]. In vitro, they increase OPC proliferation, survival, and differentiation. Notably, miR-23a-5p is highly enriched in M2-EVs and plays a key role in supporting OPC maturation and survival. Knocking down miR-23a-5p in M2-EVs reversed these beneficial effects both in vitro and in vivo. Luciferase reporter assay confirmed that miR-23a-5p directly targets Olig3 [109]. Another study identified that M2 exosomes containing high levels of miR-672-5p effectively suppress the AIM2/ASC/caspase-1 signaling pathway by inhibiting AIM2 activity, thereby reducing neuronal pyroptosis. This protective mechanism contributes to improved functional recovery in mice with SCI [110]. Finally, M2 exosomes are enriched with miR-135a-5p, which plays a neuroprotective role by regulating the TXNIP/NLRP3 signaling pathway. miR-135a-5p decreases NLRP3 expression by targeting TXNIP, leading to reduced neuronal autophagy and ischemic brain injury. Overexpression of miR-135a-5p enhances neuronal proliferation, suppresses apoptosis, and downregulates autophagy-related proteins. Furthermore, M2 exosomes effectively deliver miR-135a-5p to neuronal cells, inhibiting TXNIP expression and thereby suppressing NLRP3 inflammasome activation. These findings highlight the potential of miR-135a-5p-enriched M2-EVs as a therapeutic strategy for IBI [111] (Table 3).

**Table 3:** Studies that have specifically evaluated MDEVs and ODEVs as well as their non-coding RNA cargos in neuropathologic conditions

ncRNA cargo	EV source (ncRNA detection method)	Proposed biological function (selection reason)	Functional evaluation	EV extraction	Disease relation (~ FC)	References
miR-124-3p	CD11b-positive EVs from mouse brain, mouse BV2 cells (microarray with qRT-PCR validation)	Targets Rela/ApoE signaling pathway and promotes the Aβ-proteolytic breakdown (significantly abundant, with confirmed group differences)	Taken up by neurons, gain/loss-of-function, 3′-UTR luciferase, and ChIP-PCR analysis	UCF (Kit)	TBI (12.5-FC↑)	[99]
miR-6096-5p	CD11b-positive EVs from mouse brain (microarray)	NC	NC		TBI (6.5-FC↑)	
miR-155	Mouse BV2 cells (qRT-PCR)	Inhibits Rheb-mTOR signaling (prior literature)	Taken up by neurons, gain-of-function, and 3′-UTR luciferase analysis	UCF	Brain heat stress (2-FC↑)	[100]
miR-615-5p	Mouse SIM-A9 cells (miRNA-seq with qRT-PCR validation)	Induced by LPS, binds to MYRF, and inhibits OPC maturation (significantly abundant, confirmed group differences)	Taken up by OPCs, gain/loss-of-function, 3′-UTR luciferase, and in silico analysis	UCF (Kit)	MS (NC↑)	[101]
Let7b-5p	Mouse BV2 cells (TaqMan® qRT-PCR)	Binds to HMGB1 and induces neurotoxicity by TLR7 activation (prior literature)	Taken up by neurons, and RIP analysis	UCF	EIN (4-FC↑)	[102]
miR-146a-5p	Rat primarily microglia (Exicon® qRT-PCR)	Regulates the expression of Syt1 and Nlg1 (significantly abundant, with confirmed group differences)	Taken up by neurons, gain-of-function, and 3′-UTR luciferase analysis	UCF (Kit)	MS (FC > 2↑)	[103]
miR-223		Targets GluR2	NC			
mir-181a		Targets GluR2 and GluN2B				
miR-146a-5p	Mouse BV2 cells, serum-derived exosomes of CUMS rats (qRT-PCR)	Suppresses neurogenesis by targeting the KLF4/p-STAT3/CDKL5 pathway (prior literature, significantly abundant, with confirmed group differences)	Taken up by neurons, gain-of-function, and 3′-UTR luciferase analysis	UCF	CUMS (2-FC↑)	[104]
miR-122-5p	Serum-derived exosomes of CUMS rats (qRT-PCR)	NC	NC		CUMS (1.5-FC↑)	
miR-187-3p					CUMS (1.5-FC↑)	
miR-124-5p	Mouse BV2 cells (TaqMan® qRT-PCR)	Maintains brain homeostasis through Glu balance (prior literature)	Taken up by astrocytes, and gain-of-function	UCF	Glioma (NC↓)	[105]
miR-124-3p	Mouse BV2 cells (microarray with qRT-PCR validation)	Targets PDE4B, and inhibits the activity of mTOR signaling (significantly abundant, with confirmed group differences)	Taken up by neurons, gain-of-function, and 3′-UTR luciferase and in-silico analysis	UCF	TBI (8-FC↑)	[106]
miR-125a-5p	Mouse BV2 cells (microarray)	NC	NC		TBI (6-FC↑)	
miR-434-3p					TBI (6-FC↑)	
miR-335-5p					TBI (6-FC↑)	
miR-495-3p					TBI (5-FC↑)	
miR-128-3p					TBI (5-FC↑)	
miR-138-5p					TBI (5-FC↑)	
miR-218-5p					TBI (5-FC↑)	
miR-127-3p					TBI (4-FC↑)	
miR-376b-3p					TBI (4-FC↑)	
miR-335-3p					TBI (4-FC↑)	
miR-382-5p					TBI (3-FC↑)	
miR-100-5p					TBI (3-FC↑)	
miR-137	Mouse BV2 cells (RNA-seq with qRT-PCR validation)	Targets the Notch1 signaling pathway (prior literature, significantly abundant, with confirmed group differences)	Taken up by neurons, gain/loss-of-function, and 3′-UTR luciferase and in-silico analysis	UCF	IBI (7-FC↑)	[107]
miR-151-3p	Mouse primary microglia (qRT-PCR)	Attenuates apoptosis via regulating the p53/p21/CDK1 pathway (prior literature, significantly abundant, with confirmed group differences)	Taken up by neurons, gain/loss-of-function, 3′-UTR luciferase, and in-silico analysis	UCF	SCI (15-FC↑)	[108]
miR-23a-5p	Mouse BV2 cells (RNA-seq with qRT-PCR validation)	Induced by IL-4, promotes white matter repair and functional recovery (significantly abundant, with confirmed group differences)	Taken up by OPCs, gain/loss-of-function, and 3′-UTR luciferase	UCF	Stroke (9-FC↑)	[109]
miR-151-5p		NC	NC		Stroke (8-FC↑)	
miR-30b-3p					Stroke (6-FC↑)	
miR-501-5p					Stroke (4-FC↑)	
miR-222-3p					Stroke (4-FC↑)	
miR-365-2-5p					Stroke (4-FC↑)	
miR-221-3p					Stroke (4-FC↑)	
miR-129-5p					Stroke (4-FC↑)	
miR-155-5p					Stroke (4-FC↑)	
miR-744-5p					Stroke (4-FC↑)	
miR-423-3p					Stroke (3-FC↑)	
miR-672-5p	Mouse BV2 cells (qRT-PCR)	Inhibits the AIM2/ASC/Caspase-1 signaling pathway-mediated neuronal pyroptosis (prior literature)	Taken up by neurons, gain/loss-of-function, and 3′-UTR luciferase	UCF	SCI (NC↑)	[110]
miR-135a-5p	Mouse BV2 cells (qRT-PCR)	Inhibits TXNIP expression and activation of NLRP3 inflammasomes (prior literature)	Taken up by neurons, gain/loss-of-function, and 3′-UTR luciferase	UCF	IBI (NC↑)	[111]
miR-9	Mouse Oli-neu cells (qRT-PCR)	Reduces the expression of neuronal doublecortin (prior literature)	NC	UCF	NGDs (NC↑)	[115]
miR-19						

UCF, Ultracentrifugation; DGUC, Differential gradient ultracentrifugation; NGDs, Neurodegenerative Diseases; IBI, Ischemic brain injury; EIN, ethanol-induced neurotoxicity; HGCL, Human glioma cell lines; SCI, Spinal cord injury; FC, Fold change compared to control; NC, Not clarified. The highlighted wells were included in the bioinformatics analysis as they exhibited 100% similarity with human sequences

## ODEVs and their ncRNA content

Oligodendrocytes facilitate electric impulse propagation by ensheathing axons with a CNS myelin sheath. This myelination process involves intense communication between oligodendrocytes and neurons, which is essential for maintaining axonal integrity throughout life. Oligodendrocytes release EVs containing galactocerebroside, sulfatide, and cholesterol-essential components of oligodendroglial lipid rafts, defining characteristic myelin lipids [112]. These ODEVs exhibit a distinct proteomic profile, including marker proteins (Alix, Tsg101, Flotillin-1), ubiquitous tetraspanins, chaperones, and specific myelin proteins (PLP, MAG, MOG). Markedly, ODEVs also contain enzymes such as sirtuin-2, peroxiredoxins, dihydropyrimidinase-related proteins, and glycolytic enzymes (glyceraldehyde-3-phosphate dehydrogenase, pyruvate kinase, α-enolase) [113]. Frühbeis et al. demonstrated that the secretion of ODEVs mediated by Ca^2+^ influx through oligodendroglial *N*-methyl-*D*-aspartic acid and AMPA receptors is stimulated by activity-dependent glutamate release from neurons [114].

### miRNAs in ODEVs

The OPC line, Oli-neu, actively secretes EVs enriched with miRNA-9 and miRNA-19a. These miRNAs are predicted to target Doublecortin, a key protein involved in neuronal migration and differentiation [115]. Upon transfer to neurons, the EV-contained miRNAs significantly downregulate Doublecortin expression, potentially influencing neurodevelopmental processes. This suggests that the OPC-derived EVs regulate neuronal maturation and plasticity through miRNA-mediated post-transcriptional modifications. Further investigation into the mechanisms of ODEV-mediated miRNA transfer may provide insights into the OPC–neuron communication and its implications for neurodevelopment and repair [115]. Although the significance of some ncRNAs in oligodendrocyte function has been studied [116, 117], no studies have specifically evaluated and confirmed that circRNAs and lncRNAs can be packaged into ODEVs. Only two miRNAs (miR-9 and miR-19) have been confirmed in this regard (Table 3).

## Selection of cell-enriched, BDEV-associated ncRNAs for further analyses

**Fig. 4:**
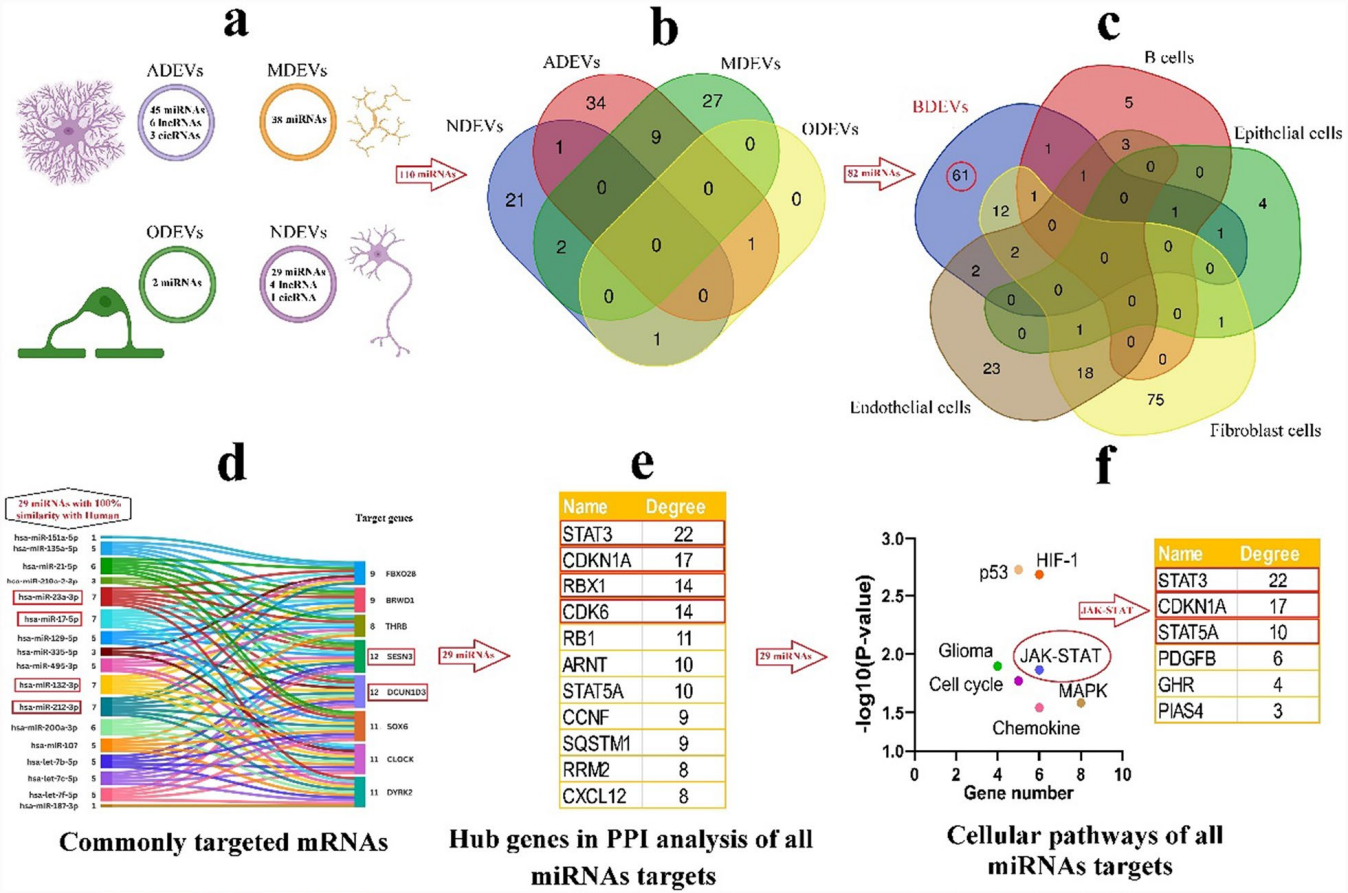
Variability in ncRNA distribution across BDEVs. **a** ADEVs exhibit the greatest diversity, containing 45 confirmed miRNAs, 3 circRNAs, and 6 lncRNAs, followed by NDEVs, which contain 29 confirmed miRNAs, one circRNA, and four lncRNAs. **b** Among the 110 confirmed BDEV miRNAs, 34 were exclusive to ADEVs, 21 to NDEVs, and 27 to MDEVs. **c** Further screening of these 82 miRNAs identified 61 miRNAs enriched in BDEVs and absent in EVs from other cell types. **d** Next, human miRNAs and animal miRNAs with 100% sequence similarity to human miRNAs were selected, narrowing the final list to 29 miRNAs for further analysis. Eight mRNAs were commonly targeted by 17 miRNAs, with *SESN3* and *DCUN1D3* being the most targeted, each interacting with 12 miRNAs. Four other mRNAs were targeted by at least seven miRNAs. **e** PPI network analysis of all target genes identified *STAT3* and *CDKN1A* as the most interactive hub genes. **f** Pathway analysis of these hub genes highlights the involvement of HIF-1, p53, and JAK-STAT signaling pathways. Further analysis of the JAK-STAT pathway PPI network confirmed *STAT3* and *CDKN1A* as key hub genes

We identified 29 miRNAs, 4 lncRNAs (lncRNA-POU3F3, lncRNA-ENST000532430, lncRNA-CAND1.11, and lncRNA-EPS15L1), and 1 circRNA (CircOGDH) in NDEVs (Table 1). Among the detected miRNAs, three families (miR-181, miR-9, and Let-7) were reported in one study [60]. However, only the most relevant members with the most significant expression changes are included in Table 1. Additionally, 4 miRNAs (PC-5p-149427_24, PC-5p-139289_26, PC-3p-38497_124, and PC-3p-275123_15) were excluded from further analysis as they were not found in available databases (Table 1). In ADEVs, 45 miRNAs (for Let-7f and miR-107, both arms −3/5p were included since the exact form was unclear in the data), 6 lncRNAs (lncRNA-NKILA, lncRNA-ATB, lncRNA-aHIF, lncRNA-CCAT2, lncRNA-POU3F3, and lncRNA-49Rik), and 3 circRNAs (circSHOC2, circRNA-ATP8B4, and circ_0012381) have been identified (Table 2). Lastly, in MDEVs, 38 miRNAs have been detected, while ODEVs contained only 2 miRNAs, with no confirmed detection of lncRNAs or circRNAs in either (Table 3). This suggests that BDEVs contain a diverse range of ncRNAs (miRNAs, lncRNAs, and circRNAs). The type and amount of ncRNAs present in different brain cells depend on the cell type, physiological conditions, and developmental stage. Among the ncRNAs detected in BDEVs, ADEVs had the highest variety with 54 different ncRNAs (Fig. 4a). Some of the ncRNAs, i.e., miRNA-26a-5p, miRNA-155-5p, miRNA-223, and miRNA-19a, are also detected in other BDEVs. In addition, NDEVs exhibit the second greatest diversity with 34 different ncRNAs (Fig. 4a). NDEV miRNAs, including miRNA-9, miRNA-124-3p, miRNA-181a-5p, and miRNA-26a-5p, are also identified in other BDEVs. Remarkably, MDEVs and ODEVs do not have any confirmed lncRNAs or circRNAs. Moreover, miRNA-181a-5p, miRNA-223-3p, miRNA-155, and miRNA-124-3p detected in MDEVs are also identified in other BDEVs. In contrast, the ncRNA content of ODEVs is still poorly characterized, with only two miRNAs—miR-9 and miR-19a—identified so far. Notably, both are also present in other types of BDEVs, suggesting they are not specific to ODEVs. In the initial step, we compared differentially expressed miRNAs across various cell groups to eliminate redundantly reported and non-cell-enriched miRNAs. As shown in Fig. 4b, we selected 21 NDEV-enriched miRNAs, 34 ADEV-enriched miRNAs, and 27 MDEV-enriched miRNAs for further analysis (Fig. 4b). This summary underscores the diversity and overlap of ncRNAs in BDEVs, highlighting the potential to carry distinct yet intersecting sets of molecular information across different brain cell types.

Next, the 82 selected miRNAs were subjected to further screening (Fig. 4c). For cell specificity analysis, we focused on B cell-, mesenchymal stem cell-, epithelial-, fibroblast-, mast cell-, and endothelial cell-derived EV miRNAs. As a result, 61 cell-enriched BDEV miRNAs were selected for future analysis (Fig. 4c). Remarkably, no overlapping miRNAs were found between our dataset and mesenchymal stem cell- or mast cell-derived EV miRNAs. Consequently, these categories are not shown in the Venn diagram (Fig. 4c). In the following step, the orthologs of these miRNAs in humans were identified using the MirGeneDB 3.0 database (https://mirgenedb.org/). Additionally, the sequence similarity between animal miRNAs and their human orthologs was evaluated using NCBI's global alignment tool. Our analysis revealed that 50 out of the 61 miRNAs had human orthologs, and among them, 29 miRNAs with 100% sequence similarity were selected for further analysis (Fig. 4d). Markedly, of these 29 miRNAs, 5 were from rats, 3 from humans, and others from mouse-derived cells (highlighted in Tables 1, 2 and 3).

Since the same screening analysis performed for miRNAs, such as determining cell-exosomal specificity, identifying orthologs, and assessing sequence similarity to human sequences, could not be applied to circRNAs and lncRNAs, we selected all circRNAs and lncRNAs with available complete sequences for further analysis. Using this approach, we identified three circRNAs (mouse OGDH, mouse SHOC2, and human 0012381) and six human lncRNAs (CAND1.11, lncRNA-NKILA, lncRNA-ATB, lncRNA-aHIF, lncRNA-CCAT2, and lncRNA-POU3F3) for future studies.

## Targets, hub genes, and cellular pathways of BDEV-derived miRNAs

After evaluating experimentally confirmed EVs and applying selection criteria, 26 animal miRNAs with 100% sequence similarity to humans, as well as 3 human miRNAs, were selected. The common target genes (mRNAs) of these 29 miRNAs were predicted. Two genes, Sestrin 3 (*SESN3*) and Defective in Cullin Neddylation 1 Domain Containing 3 (*DCUN1D3*), were identified to be the most highly targeted, each being regulated by 12 miRNAs (Fig. 4d). Additionally, miRNA-23a-3p, miRNA-17-5p, miRNA-132-3p, and miRNA-212-3p demonstrate the highest interaction with common genes, each engaging in seven interactions (Fig. 4d).

A PPI network was established and analyzed for all target genes. Four key genes including Signal Transducer and Activator of Transcription 3 (*STAT3*), Cyclin-Dependent Kinase Inhibitor 1 A (*CDKN1A*), RING-Box Protein 1 (*RBX1*), and Cyclin-Dependent Kinase 6 (*CDK6*) exhibited the highest interaction degree among the hub genes (Fig. 4e). The cellular pathways influenced by the 29 selected miRNAs were analyzed, revealing that the HIF-1, p53, Janus kinase-signal transducer and activator of transcription (JAK-STAT), and MAPK (mitogen-activated protein kinase) pathways are the most significantly associated pathways (Fig. 4f). Finally, analysis of PPIs for genes involved in the JAK-STAT pathway revealed *STAT3*, *CDKN1A*, and Signal Transducer and Activator of Transcription 5 A (*STAT5A*) as key hub genes (Fig. 4f). Noteworthy is that the mRNAs of these genes are principal targets of the detected miRNAs (Fig. 4e). These findings suggest that BDEV-associated miRNAs under pathological conditions may regulate critical mRNAs, potentially contributing to neuropathological processes. They present new avenues for further exploration in neurodegenerative disease research.

## Targets, hub genes, and cellular pathways of BDEV-enriched miRNAs

**Fig. 5:**
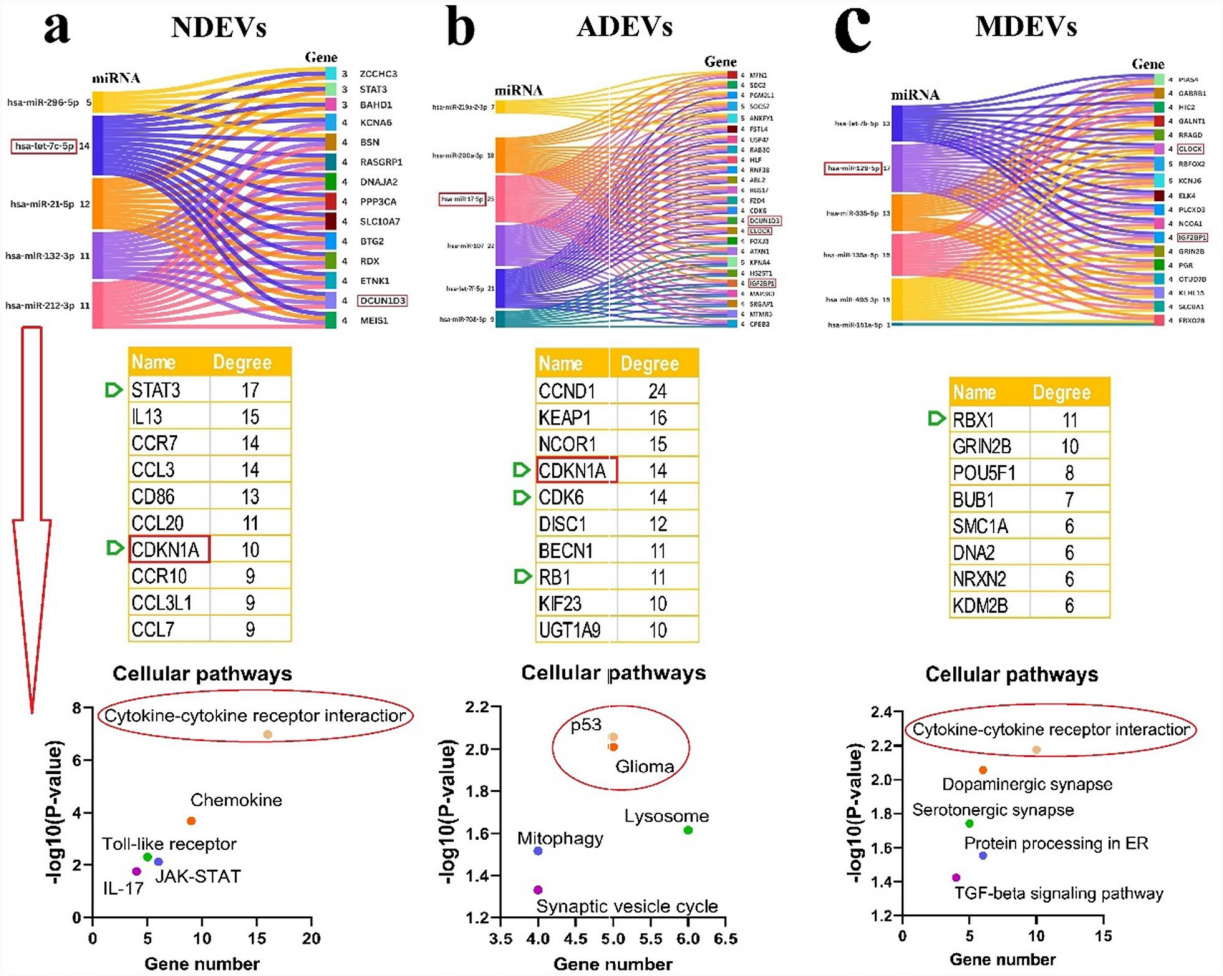
Identification of targets of BDEV miRNAs. Common gene targets of miRNAs were predicted in a cell-specific manner, followed by PPI network and cellular pathways analysis. **a** In NDEVs, five miRNAs targeted 14 common genes, with Let7-c-5p interacting with all of them, while *DCUN1D3* was also targeted by ADEV-derived miRNAs. **b** In ADEVs, six miRNAs targeted 25 common genes, with miR-17-5p interacting with all, while *CLOCK* and *IGF2BP1* were also targeted by MDEV-derived miRNAs. **c** In MDEVs, six miRNAs targeted 18 common genes, with miR-129-5p interacting with 17 of them. PPI network analysis identified STAT3 as the top hub protein for NDEV miRNAs, and CCND1 emerged as the top hub protein for ADEV miRNAs. CDKN1A is among the top 10 hub proteins for both NDEV and ADEV miRNAs. For MDEVs, RBX1 was the predominant hub protein. Cellular pathways associated with the hub proteins include inflammatory-related pathways predominantly involved in NDEVs (**a**), glioma-related pathways in ADEVs (**b**), and neuronal and cytokine-related pathways in MDEVs (**c**)

Five NDEV-enriched miRNAs target 14 common mRNA genes (Fig. 5a). Among them, *DCUN1D3* is also a target of ADEV-enriched miRNAs (Fig. 5b). Particularly, miRNA-let-7c-5p interacts with all common genes (Fig. 5a). A PPI network analysis of NDEV miRNA target genes identified *STAT3* as a key hub gene (Fig. 5a), aligning with its previously identified role as a hub gene targeted by all miRNAs (Fig. 4e). Additionally, *CDKN1A* was identified as a shared hub gene between NDEV- and ADEV-derived miRNA targets (Fig. 5b). Finally, an evaluation of the cellular pathways associated with these hub genes revealed that cytokine-cytokine receptor interactions and chemokine signaling pathways are most significantly associated with the NDEV-enriched miRNAs, with the JAK-STAT pathway being a key component (Fig. 5a).

Six miRNAs derived from ADEVs were found to target 25 common mRNA genes (Fig. 5b). Among them, Circadian Locomotor Output Cycles Kaput (*CLOCK*) and Insulin-Like Growth Factor 2 mRNA-Binding Protein 1 (*IGF2BP1*) are also targeted by MDEV-derived miRNAs (Fig. 5c). Notably, miRNA-17-5p exhibits the highest interaction, targeting all 25 common genes. A PPI network was constructed and analyzed for all target genes of ADEV miRNAs, identifying Cyclin D1 (*CCND1*) as a major hub gene (Fig. 5b). Additionally, *CDK6* and Retinoblastoma 1 (*RB1*) were previously identified as key hub genes targeted by all miRNAs (Fig. 4e). Finally, pathway analysis of these top hub genes revealed glioma, p53, and ribosome pathways as key regulatory pathways. The top hub genes are also involved in synaptic vesicle cycle and mitophagy (Fig. 5b).

Six miRNAs derived from MDEVs were identified to target 18 common mRNA genes (Fig. 5c). Among these, *CLOCK* and *IGF2BP1* were also targeted by ADEV-derived miRNAs as described previously (Fig. 5b). Importantly, miRNA-129-5p demonstrated the highest interaction, targeting 17 common genes (Fig. 5c). A PPI network analysis of all target genes of MDEV-derived miRNAs identified *RBX1* as a key hub gene. *RBX1* was previously also identified as a key hub gene targeted by all miRNAs (Fig. 4e). Lastly, pathway analysis of these top hub genes highlighted cytokine-cytokine receptor interactions, dopaminergic synapse, and serotonergic synapse as crucial regulatory pathways, along with additional roles in ER protein processing and TGF-β signaling (Fig. 5c).

## Targets, hub genes, and cellular pathways of BDEV-enriched LncRNAs and circRNAs

**Fig. 6:**
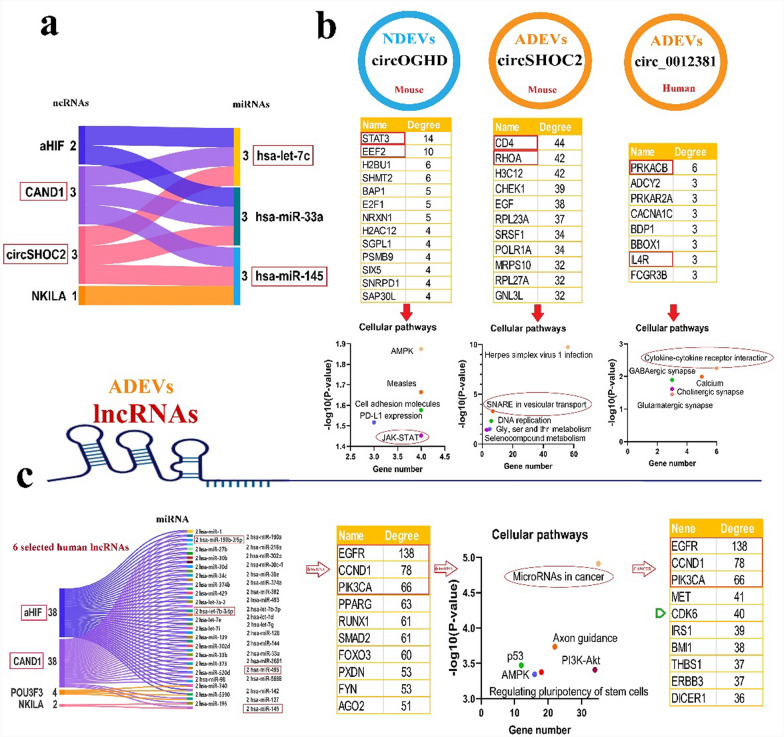
Target evaluation of lncRNAs and circRNAs in BDEVs.** a** A comprehensive miRNA-target prediction analysis was conducted for three selected circRNAs (circSHOC2, circOGHD, and circ_0012381) and six selected lncRNAs (CAND1.11, lncRNA-NKILA, lncRNA-ATB, lncRNA-aHIF, lncRNA-CCAT2, and lncRNA-POU3F3) with available complete sequences. Particularly, two of these miRNAs, let-7c and miR-145, are also contained in BDEVs. **b** PPI network was constructed for miRNAs targeted by each circRNA. For circOGHD, the top hub proteins identified are STAT3 and EEF2, with the JAK-STAT signaling pathway showing the most significant relevance. For circSHOC2, the top hub proteins are CD4 and RHOA, with the SNARE vesicular transport pathway showing notable relevance. For circ_0012381, PRKACB and IL4R are identified as key hub, with the cytokine-cytokine receptor interaction pathway showing the most significant relevance.** c** Common miRNA-target predictions were performed for the six selected lncRNAs. Among these, only four lncRNAs share common miRNA interactions. Notably, CAND1 and aHIF were predicted to target a significant number of 38 miRNAs, among which let-7b, miR-145, miR-190b, and miR-495 have been detected in BDEVs. PPI network constructed for miRNAs targeted by these lncRNAs reveal that *EGFR*, *CCND1*, and *PIK3CA* are three top hub genes. These hub genes are significantly enriched in key cellular pathways, including miRNAs in cancer, axon guidance, and the PI3K-Akt signaling pathway. Interestingly, when focusing on miRNAs involved in cancer, the same three hub proteins (EGFR, CCND1, and PIK3CA) were once again highlighted, emphasizing their crucial role in both cancer-related and neuroinflammatory regulatory networks

Among the nine identified circRNAs and lncRNAs, one circRNA (mouse SHOC2 in ADEVs) and three human lncRNAs (aHIF and NKILA in ADEVs, and CAND1 in NDEVs) target three common miRNAs (Fig. 6a). Among these miRNAs, Let-7c and miR-145 are confirmed to be expressed in NDEVs and ADEVs, respectively (Tables 1 and 2). A PPI network was constructed and analyzed for all target miRNAs of the three selected circRNAs (Fig. 6b). For circOGDH, *STAT3* is the most significant hub gene (Fig. 6b), which is also a hub gene targeted by BDEV- (Fig. 4e) and NDEV-derived miRNAs (Fig. 5a). Pathway analysis of these key hub genes revealed AMPK, JAK-STAT, and cell adhesion molecules as major regulatory pathways, with additional involvement in PD-L1 (programmed death-ligand 1) signaling, emphasizing its relevance in immune regulation (Fig. 6b). Additionally, the PPI network analysis for circSHOC2 targets identified CD4, a glycoprotein co-receptor for the T-cell receptor, as a key hub gene for the first time (Fig. 6b). Pathway analysis of these hub genes revealed SNARE-mediated vesicular transport, DNA replication, and selenocompound metabolism as significantly associated pathways (Fig. 6b). Furthermore, the PPI network analysis for circSHOC2 targets identified *PRKACB* (protein kinase cAMP-activated catalytic subunit beta), a crucial component of the cAMP signaling pathway, as a key hub gene for the first time (Fig. 6b). Pathway analysis of these hub genes highlighted cytokine-cytokine receptor interaction, GABAergic synapse, calcium signaling, cholinergic synapse, and glutamatergic synapse pathways (Fig. 6b).

Among the six selected human lncRNAs, four (aHIF, POU3F3, and NKILA in ADEVs and CAND1 in NDEVs) were found to target 41 miRNAs (Fig. 6c). No single miRNA was exclusively targeted by these lncRNAs. However, among these miRNAs, let7-c, miR-145, miR-190, and miR-495 (belonging to the final set of 29 selected miRNAs) were confirmed to be expressed in NDEVs, ADEVs, ADEVs, and MDEVs, respectively (Tables 1, 2 and 3). Lnc-aHIF and CAND1 exhibited a strong regulatory potential by targeting a substantial number of miRNAs, with a total of 38 miRNAs identified as their targets (Fig. 6c). This suggests that these lncRNAs may be crucially involved in post-transcriptional gene regulation, potentially influencing various cellular processes by modulating miRNA activity. Their widespread interaction with multiple miRNAs highlights their significance in broader regulatory networks, warranting further investigation into their functional impact on gene expression and cellular pathways.

Additionally, a PPI network analysis of the target miRNAs associated with six selected human lncRNAs identified the epidermal growth factor receptor (*EGFR*), *CCND1*, and phosphatidylinositol-4,5-bisphosphate 3-kinase catalytic subunit alpha (*PIK3CA*) as the top three hub genes (Fig. 6c). Of particular interest, *CCND1* is also a primary hub gene targeted by miRNAs derived from ADEVs (Fig. 5c). Further pathway enrichment analysis revealed involvement of hub genes in several critical cellular pathways, including miRNAs in cancer, axon guidance, p53 signaling, PI3K-AKT signaling, AMPK signaling, and regulation of pluripotency in stem cells (Fig. 6c). Among these pathways, the p53 pathway is also a key cellular pathway influenced by all 29 selected miRNAs (Fig. 4f) and ADEV-associated miRNAs (Fig. 5b). The evaluation of PPI networks related to miRNAs involved in cancer further reinforced the central role of *EGFR*, *CCND1*, and *PIK3CA*, as they consistently emerged as the top three hub genes (Fig. 6C).

## Conclusion

There is ongoing research aiming to identify unique ncRNA markers that can reliably distinguish cell-type-specific EVs (CTEVs) from those originating in the brain and released into the blood. This task is complex and challenging due to the heterogeneous nature of EVs and the overlapping ncRNAs among vesicles from different cells. Identifying and confirming enriched ncRNA loading in CTEVs is crucial for predicting their function and establishing a unique epigenetic signature for each CTEV. In vitro studies of sEVs derived from specific cells can help evaluate their cargos, but replicating the pathological conditions that occur at the multicellular level remains difficult. The comprehension gaps surrounding the mechanisms of CNS diseases, coupled with challenges in selecting BDEVs and scalability limitations, pose significant obstacles to their clinical application. Distinguishing BDEVs in the blood and evaluating their ncRNA cargos have significant implications for understanding the pathophysiology of neurodegeneration, brain injury, and other neurological disorders. Specific ncRNAs (mostly miRNAs) enriched in BDEVs may serve as potential biomarkers for determining the origin of sEVs. For instance, through a combination of experimental evaluations and database analyses, we identified 61 unique miRNAs specifically packaged into BDEVs. However, further complementary experiments are necessary to confirm their exclusivity and ensure that they are not present in other EV subtypes.

The target hub genes regulated by BDEV-derived miRNAs are involved in key biological processes, particularly inflammation (STAT3, CDKN1A, and the JAK-STAT signaling pathway) and cell cycle regulation (RBX1, CDK6, and the p53 signaling pathway). This finding suggests that, under certain pathological conditions, brain cells may collectively express a shared set of cell cycle regulatory and anti-inflammatory miRNAs via their sEVs to mitigate neuroinflammation. In this context, different BDEV subtypes may have specialized roles in regulating neuroinflammatory responses. NDEVs and MDEVs appear to be more directly involved in neuroinflammation by modulating cytokine-cytokine receptor interactions and chemokine signaling. ADEVs seem to act as a major factor in cell cycle regulation, primarily through the p53 pathway, which is essential for cellular homeostasis and neuroprotection. The intricate intercommunication between BDEVs and MDEVs becomes even more evident with the identification of dopaminergic and serotonergic synapse pathways as key cellular pathways influenced by MDEV-derived miRNAs. These findings highlight a potential mechanism by which EV-mediated miRNA transfer contributes to the regulation of neurotransmitter systems.

On the other hand, circOGDH derived from NDEVs was found to target anti-inflammatory miRNAs, which predominantly act as sponges for *STAT3* mRNA. This suggests that the transfer of circOGDH via NDEVs to recipient cells could lead to upregulation of STAT3, ultimately influencing the JAK/STAT signaling pathway, a key pathway of neuroinflammation. Moreover, ADEV-derived circSHOC2 may also contribute to neuroinflammation by targeting miRNAs that sponge *CD4* and *RHOA* mRNAs. Given the roles of CD4^+^ T cells in neuroimmune activation and RHOA in microglial migration and BBB integrity, ADEV-derived circSHOC2 may promote neuroinflammation, particularly in diseases such as MS, AD, and PD. Additionally, ADEV-derived circ_0012381, through its interaction with specific miRNAs, may act as a sponge for *PRKACB* and *IL4R* mRNAs. Since PRKACB regulates inflammatory signaling and microglial homeostasis, and IL4R is involved in anti-inflammatory responses, the differential expression of these circRNAs within BDEVs may provide insights into cell-specific contributions to neuroinflammation via circRNA–miRNA–mRNA interactions, with possible therapeutic implications for neurodegenerative and neuroimmune disorders.

In the context of lncRNAs, six selected lncRNAs were found to target miRNAs that act as sponges for three top hub genes: *EGFR*, *CCND1*, and *PIK3CA*. These genes are intricately involved in neuroinflammation, though their specific contributions vary depending on cell type, signaling pathways, and pathological conditions. Notably, most of these lncRNAs have been previously reported in GDEVs, which aligns with their association with miRNAs in cancer and their involvement in the p53 signaling pathway, a crucial regulator of cell cycle arrest, apoptosis, and inflammatory responses. The findings suggest that these lncRNA–miRNA–mRNA interactions can modulate key signaling cascades involved in glioblastoma, neurodegeneration, and immune responses. Further investigations into their cell-type-specific roles may reveal new therapeutic targets for cancer and neuroinflammatory disorders.

Our findings suggest that the 29 selected miRNAs, which interact with circRNAs and lncRNAs, may have their regulatory effects modulated or inhibited by these non-coding RNAs, especially when they are co-expressed within the same BDEVs. Conversely, if these regulatory RNAs are expressed in different types of BDEVs, they may exert distinct regulatory effects on cellular environments, potentially influencing intercellular communication and gene expression dynamics.

There are distinct, cell-type-specific patterns of ncRNA expression within BDEVs, highlighting complex regulatory roles of these molecules in intercellular communication under physiological and pathological conditions. The Let-7 family is a notable example, which exhibits differential expression across BDEVs of various origins and under different disease states. Human NDEVs show enrichment in Let-7c-5p, implicated in the modulation of neuroglial interactions during neurodegenerative processes and is preferentially taken up by astrocytes. In contrast, ADEVs also contain Let-7f-3p and Let-7f-5p, which are involved in inflammatory signaling and can be internalized by neurons. MDEVs are notably enriched with Let-7b-5p, a key regulator of neuroinflammation with neuronal uptake. There is currently a lack of evidence for Let-7 family expression in ODEVs, leaving their epigenetic contribution to white matter homeostasis less defined.

Furthermore, disease-specific alterations in BDEV-associated ncRNAs are increasingly evident in animal models. In AD, a human study reported a marked decrease in NDEVs carrying miR-132-3p and miR-212-3p, both of which are crucial for synaptic plasticity and neuronal integrity [58]. In ALS animal models, studies have revealed a reduction of NDEVs containing miR-124-3p, a miRNA involved in neuronal differentiation and inflammation [61]. Elevated levels of miR-21a-5p in NDEVs have been identified as a potential biomarker of TBI and broader neurodegenerative changes. ADEVs also demonstrate disease-associated shifts in miRNA profiles [63]. In ischemic brain injury and hypoxic-ischemic encephalopathy, increased levels of miR-190b-5p and miR-17-5p have been observed in ADEVs. Conversely, miR-200a-3p levels are reduced in ADEVs in PD, suggesting a loss of regulatory control over oxidative stress and apoptosis [83]. MDEVs reflect inflammatory and immune-mediated pathologies. For example, miR-615-5p is elevated in MDEVs in MS, and miR-151-5p is implicated in stroke-related pathology [101]. On the other hand, in TBI models, increased expression of miR-335-5p and miR-495-3p in MDEVs is associated with modulation of neuroinflammatory responses and neuronal repair. These findings collectively emphasize that the ncRNA cargos of BDEVs are not only highly cell-specific but also dynamically modulated in response to various neuropathological conditions. This underscores the potential of BDEVs as minimally invasive biomarkers and therapeutic delivery systems in CNS diseases. Importantly, there is a critical need for translational studies to validate these observations in human patients, in order to ensure the clinical applicability of BDEV-based diagnostics and therapeutics. While our findings provide a strong basis for understanding specific miRNA cargos of BDEVs, they should be interpreted as an initial framework rather than definitive evidence. Future research should focus on validating these miRNAs using advanced techniques such as high-throughput sequencing, single-vesicle analysis, and functional assays to determine their biological significance and regulatory roles. Additionally, investigating the mechanisms governing their selective packaging into BDEVs could offer deeper insights into cell-to-cell communication and disease pathogenesis.

## Limitations

Several challenges remain in this field. One major limitation is the discrepancy between transcriptomic predictions and functional validation. While high-throughput sequencing has identified numerous ncRNAs within BDEVs, only a small subset has undergone further experimental validation through RT-PCR and functional studies. This limits our understanding of the biological significance of many ncRNAs detected in transcriptomics datasets. Additionally, heterogeneity in EV populations, as well as differences in isolation protocols and experimental conditions, pose challenges in standardizing findings across studies. Furthermore, the mechanistic interactions between ncRNAs within EVs remain largely unexplored, making it difficult to determine how these molecular cargos work in concert to regulate gene expression in recipient cells.

A critical yet often overlooked issue is the precise localization of ncRNAs within EVs. Many studies do not distinguish whether the detected RNAs are encapsulated inside the vesicles, loosely associated with their surface, or simply contaminants from the isolation process. Additionally, while there are methods to assess RNA localization within EVs (such as RNase protection assays), they are rarely employed in most studies. This distinction is crucial, as only internalized ncRNAs are likely to be effectively delivered to target cells. Traditional EV isolation methods (such as ultracentrifugation) are widely used, but are prone to co-isolation of RNA-binding proteins and extracellular RNA fragments, making it difficult to determine whether an identified ncRNA is functionally relevant. Although more refined isolation techniques like density gradient separation can enhance EV purity, they remain underutilized in the field.

One of the most significant limitations in this field lies in translating findings from animal models to human disease contexts. The majority of studies were conducted in animal models due to the feasibility of gain- and loss-of-function experiments. Therefore, generalizing results to human pathophysiology remains a challenge. To bridge this gap, we recommend the use of human-derived neuronal and glial cell lines for validating findings in vitro. Since blood is the most accessible source of biological material from neurological patients, isolating BDEVs from blood samples and identifying their cell-specific origins should be prioritized. Where possible, the ncRNA content of BDEVs should be evaluated under disease-relevant stressors using in vitro systems. However, this strategy remains limited for disorders with complex etiologies or for diseases that lack animal models. Additionally, the absence of highly specific markers (for instance, to distinguish microglial exosomes from those of peripheral macrophages) complicates the precise identification of the cellular source of BDEVs isolated from whole blood, serum, or plasma samples. It is important to consider that exosomes exert not only endocrine effects but also paracrine and autocrine functions. Capturing these signaling modalities, particularly in animal studies, will require the development of more sophisticated analytical tools and models.

Finally, clinical use of EVs faces ethical challenges in informed consent, privacy, regulation, and accessibility. Patients must be aware of how their biofluid-derived EVs are collected, stored, and used, as they may contain sensitive genetic data. Ensuring data security and compliance with regulations like GDPR (General Data Protection Regulation) and HIPAA (Health Insurance Portability and Accountability Act) is crucial. Therapeutically, challenges include standardization, safety concerns, and potential immune responses. Additionally, high costs may limit accessibility, raising healthcare equity issues. Transparent guidelines and regulatory oversight are essential for the ethical integration of EVs into clinical practice.

## Future perspectives

To further advance our understanding of the functions of BDEV-carried ncRNAs, studies employing loss- and gain-of-function experiments, reporter assays, and in vivo models to elucidate their precise role in neuronal and glial communication are needed. Moreover, single-vesicle analysis and high-resolution profiling techniques could help uncover the diversity of EV subtypes and their enriched ncRNA signatures. Standardization of methods of EV isolation and characterization is crucial for reproducibility across studies. The sEV extraction strategy should be consistent and highly reproducible to facilitate biomarker screening and ensure reliable data interpretation. For example, the yield of sEVs isolated from blood (whole, serum, or plasma) using sequential ultracentrifugation is typically around 40% of the initial concentration. Ultracentrifugation (> 100,000 × *g*) often causes aggregation, fusion, and structural disruption of sEVs, affecting downstream analysis. Alternative techniques like density gradient centrifugation (using sucrose or iodixanol) are recommended to overcome these issues, but they are time-consuming. Molecular exclusion chromatography separates particles by size but can be influenced by column pore size, gel type, and elution rate. Polymer precipitation methods, used in commercial kits like ExoQuick (System Biosciences) and Total Exosome Isolation Reagent (Invitrogen), reduce exosome solubility, leading to precipitation. While these methods are faster and yield smaller EVs, the extraction quality can be uncertain.

Recently developed techniques offer certain advantages, including microfluidic systems, nanoscale lateral displacement arrays, acoustic separation, nanostructure-based filtration, nanophotonic chips, and dialysis membrane filtration [118–120]. For example, the immunoaffinity-based microfluidic method can isolate sEVs with high purity. However, challenges such as high cost and potential changes to the sEV phenotype during isolation pose obstacles to downstream analysis. In microfluidic systems, the small size of exosomes and the presence of similarly sized particles mean that a single separation method based on physical characteristics is often insufficient. Integrating various exosome separation methods, such as surface acoustic waves and magnetic cell sorting, into a microfluidic system could help address this issue.

Exploring the clinical applications of BDEVs offers a promising avenue to bridge the gap between basic research and translational medicine. One of the most compelling opportunities lies in the development of EV-based liquid biopsies for early detection and monitoring of neurological diseases. Given their ability to cross the BBB and carry cell-specific molecular cargos, BDEVs isolated from peripheral blood may serve as minimally invasive biomarkers that reflect real-time pathological changes within the CNS. Additionally, the therapeutic potential of EVs is gaining traction, particularly in the context of RNA-based therapeutics. Engineered EVs can be harnessed to deliver neuroprotective or gene-silencing RNAs directly to targeted brain regions or specific cell types, offering a more precise and less immunogenic alternative to traditional drug delivery methods. As technologies such as RNA cargo loading, cell-specific targeting, and vesicle engineering continue to advance, BDEVs hold immense potential not only for diagnostic innovation but also for the development of personalized treatments for neurodegenerative and neuroinflammatory disorders.

Furthermore, it is essential to use rigorous methodologies to distinguish between internalized and externally associated RNAs within EV preparations. Use of RNase protection assays, membrane-permeable dyes, and high-resolution imaging techniques should also be encouraged to confirm the intracellular localization of EV-associated ncRNAs. This will help determine whether the detected ncRNAs are biologically functional or merely experimental artifacts. Furthermore, interdisciplinary approaches integrating multi-omics technologies (e.g., transcriptomics, proteomics, and metabolomics) could provide a more holistic understanding of how EV-associated ncRNAs contribute to brain physiology and pathology. Ultimately, addressing these methodological challenges and improving experimental rigor will be key to unlocking the therapeutic potential of ncRNAs in BDEVs for neurological disorders.

## Data Availability

All supporting databases and softwares referenced in this article are openly available without restriction.

## Abbreviations

EV: Extracellular vesicle
ncRNA: Non-coding RNA
miRNA: MicroRNAs
siRNA: Small-interference RNA
circRNA: Circular RNA
LncRNA: Long ncRNA
CNS: Central nervous system
ILV: Intraluminal vesicle
MVB: Multivesicular bodies
ISEV: International Society for Extracellular Vesicles
ALS: Amyotrophic Lateral Sclerosis
AD: Alzheimer's disease
HD: Huntington's disease
PD: Parkinson's disease
MS: Multiple sclerosis
BBB: Blood–brain barrier
BDEV: Brain-derived extracellular vesicle
NDEV: Neuronal-derived extracellular vesicle
ADEV: Astrocytes-derived extracellular vesicle
MDEV: Microglia-derived extracellular vesicle
ODEV: Oligodendrocytes-derived extracellular vesicle
ESCRT: Endosomal sorting complex required for transport
CSF: Cerebrospinal fluid
NLGN3: Neuroligin 3
L1CAM: L1 cell adhesion molecule
MOG: Myelin oligodendrocyte glycoprotein
GFAP: Glial fibrillary acidic protein
MAG: Myelin-associated glycoprotein
PLP: Proteolipid protein
TUBB3: Tubulin beta 3 class III
SVZ: Sub-ventricular zone
NSCs: Neuronal stem cells
TBI: Traumatic brain injury
ASD: Autism spectrum disorder
OGDH: Oxoglutarate dehydrogenase
PTEN: Phosphatase and tensin homolog
IL: Interleukin
NF-κB: Nuclear factor κB
ATP8B4: ATPase phospholipid transporting 8B4
NKILA: NF-KappaB interacting lncRNA
NLRX1: Nod-like receptor X1
HIF-1α: Hypoxia-inducible factor-1α
CCAT2: Colon cancer-associated transcript 2
POU3F3: POU Class 3 Homeobox 3
TGFβ: Transforming growth factor beta
MTORC1: Mammalian target of rapamycin complex 1
MYRF: Myelin regulator factor
CDKN1A: Cyclin-dependent kinase inhibitor 1A
EGFR: Epidermal growth factor receptor
CCND1: Cyclin D1
CTEV: Cell type-specific EV
GLT1: Glutamate transporter-1
